# A Self‐Organized Liquid Reaction Container for Cellular Memory

**DOI:** 10.1002/advs.202512500

**Published:** 2026-01-12

**Authors:** Sukanta Mukherjee, Enrico Skoruppa, Holger Merlitz, Jens‐Uwe Sommer, Helmut Schiessel

**Affiliations:** ^1^ Cluster of Excellence, Physics of Life TU Dresden Dresden Germany; ^2^ Leibniz‐Institut für Polymerforschung Dresden Dresden Germany; ^3^ Institut für Theoretische Physik TU Dresden Dresden Germany

**Keywords:** biomolecular condensates, DNA‐assisted protein condensation, epigenetic memory

## Abstract

Epigenetic inheritance during cell division is essential for preserving cell identity by stabilizing the overall chromatin organization. Heterochromatin, the condensed and transcriptionally silent fraction of chromatin, is marked by specific epigenetic modifications that are diluted during each cell division. Here, we build a physical model, based on the formation of a biomolecular condensate, a liquid ‘droplet’, that promotes the restoration of epigenetic marks associated with heterochromatin. Heterochromatin facilitates the droplet formation via polymer‐assisted condensation (PAC). The resulting condensate serves as a reaction chamber to reconstruct the lost epigenetic marks. We incorporate the enzymatic reactions into a particle‐based simulation and monitor the progress of the heterochromatic epigenetic markers through an in silico analogue of the cell cycle. We demonstrate that the proposed mechanism is robust and stabilizes the heterochromatin domains over many cell generations. This mechanism and variations thereof might be at work for other epigenetic marks as well.

## Graphical abstract

How cells restore epigenetic information lost during replication is not known. This work proposes a mechanism based on the formation of biomolecular condensates. These condensates are induced by the chromosome itself and serve as reaction vessels for reconstructing missing epigenetic markers. The study is a proof of concept, demonstrating that epigenetic information can be reconstructed across 50 cell generations.

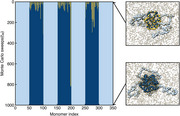

## 1 Introduction

When cells copy their genetic information in preparation for cell division, they face the challenge of also duplicating their epigenetic information. Epigenetics refers to stable gene activity‐regulating modifications beyond the primary DNA sequence that are inheritable and persist through cell divisions.^[^ ^1^, ^2^ ^]^ These modifications add an additional layer of information to the genetic layer and play an important role in defining cell types. At the molecular level, one of the mechanisms by which epigenetic information is stored concerns covalent modifications of histone proteins.^[^ ^1^, ^2^ ^]^ These proteins form the core of nucleosomes, the elementary packaging units of chromatin. In a nucleosome, about 150 base pairs (bp) of DNA—roughly its persistence length—are wrapped around an octamer of histone proteins ^[^ ^3^, ^4^ ^]^ and neighboring nucleosomes are connected by short stretches of linker DNA. Certain epigenetic marks associated with histone proteins play a vital role in differentiating the morphology of chromatin, i.e., the more open euchromatin or the less accessible heterochromatin. Therefore, cell type‐specific marks result in cell type‐specific packaging and accessibility of the genetic material.

**Figure 1:**
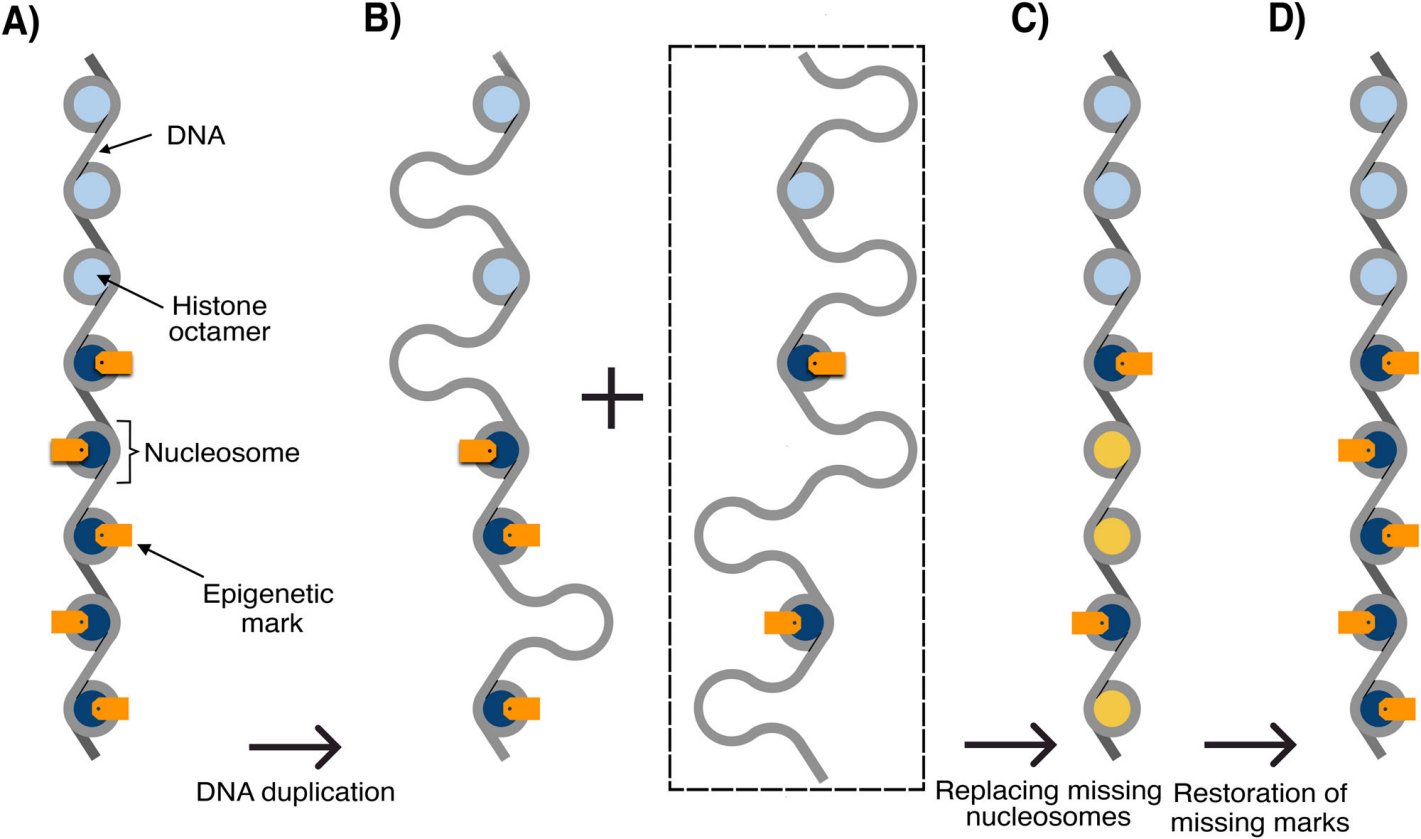
Dilution of epigenetic information during cell division: (A) A small section of a chromosome containing nucleosomes without and with marks (orange). Heterochromatin is indicated in dark blue and euchromatin in light blue. (B) As a result of DNA duplication, the string of nucleosomes along the parent DNA molecule gets distributed between the two daughter DNA molecules. This leaves half of the sites vacant. We only follow the state of the right daughter chromosome, highlighted by a dashed box. (C) Missing nucleosomes are then replaced by the addition of new initially unmethylated octamers (yellow). (D) The challenge is to re‐establish the missing epigenetic marks.

During DNA duplication, the nucleosomes are randomly distributed between the two daughter chromosomes, and vacant nucleosome positions are filled by new and unmodified nucleosomes,^[^ ^2^, ^5^ ^]^ see **Figure** **1**. Since only the old nucleosomes carry the original epigenetic marks, this process results in a “dilution” of marks by a factor of two. To prevent the loss of epigenetic information, a mechanism to reconstruct the missing marks is required.

Earlier attempts to understand how epigenetic information is established and propagated through cell divisions typically invoke scenarios where epigenetic marks spread along a 1d array of nucleosomes.^[^ ^6^, ^7^, ^8^ ^]^ For example, an important epigenetic mark is H3K9me3, a trimethylation modification on histone H3—one of the four histones that make up the histone octamer. Nucleosomes with this mark typically occur in longer blocks of many such nucleosomes (in humans, blocks have a median length of 50 nucleosomes^[^ ^9^ ^]^). There is a protein called heterochromatin protein 1 (HP1) that has a specific binding site for this mark.^[^ ^6^, ^7^, ^10^ ^]^ This led the authors of Ref. [6] to propose the following scenario: HP1 binds to nucleosomes with the H3K9‐mark, which in turn recruits an enzyme called SUV39H1 methylase; when bound to HP1, this enzyme methylates neighboring non‐methylated nucleosomes. As a result, a methylation wave propagates through the 1D chain of nucleosomes up to the point where it encounters a “boundary element”.^[^ ^6^, ^8^, ^9^ ^]^ However, the physical nature of such boundary elements remains unclear.

The missing element in this 1D picture is the 3D folding of chromosomes. Different parts of chromosomes might not be as effectively isolated from each other, and the methylation wave might jump over to a nucleosome nearby in 3D space but far away along the chain of nucleosomes. Moreover, even if a perfectly 1D mechanism existed, an inadvertently missing nucleosome in a chain of nucleosomes could completely stop the spreading of the methylation marks along the array.

This suggests that it is necessary to consider an alternative mechanism in which the restoration of the methylation marks after DNA duplication relies on the 3D structure of the chromosome. Contact maps from chromosome capture experiments provide the relevant information to this problem. Such maps highlight nucleosomes with an H3K9me3 mark to have a much higher probability of being close to other nucleosomes with this mark than to nucleosomes without the mark.^[^ ^11^ ^]^ Contact maps reveal the presence of two primary chromatin compartments: euchromatin, characterized by a relatively open structure with nucleosomes largely lacking the H3K9me3 mark, and heterochromatin, which is more densely packed and primarily composed of H3K9‐methylated nucleosomes.

In addition to accounting for the 3D chromatin structure, a physical scenario should satisfy at least the following three requirements: (A) The epigenetic state must be preserved across not only a single‐ but up to about 50 cell divisions, the Hayflick limit.^[^ ^12^ ^]^ (B) Experiments based on quantitative mass spectroscopy indicate that the reconstruction of the marks is extremely slow, of the order of 20 h.^[^ ^13^ ^]^ Any mechanism that requires processes to occur on a much shorter time scale is therefore unlikely to reflect the real system. (C) Since system properties vary with time (see below) and are vastly different across different cell types, candidate mechanisms should be robust against parameter changes.

A substantial body of literature already exists exploring various aspects of epigenetics from a physics perspective.^[^ ^14^, ^15^, ^16^, ^17^, ^18^, ^19^, ^20^, ^21^, ^22^, ^23^, ^24^, ^25^, ^26^, ^27^, ^28^, ^29^ ^]^ Of those, two simulation studies^[^ ^24^, ^28^ ^]^ make explicit use of the 3D structure of chromosomes to reconstruct the marks lost during duplication. However, the first study^[^ ^24^ ^]^ does not meet the requirements listed above. In this study, chromatin is modelled as a highly coarse‐grained copolymer where heterochromatin regions compact in the presence of HP1. Subsequently, the chromosome conformation is frozen, and methylation marks are removed. Still frozen, epigenetic marks are reassigned to individual monomers with probabilities based on the density of HP1 proteins in the vicinity of a given monomer. Only after the catalytic process is complete, the polymer is free to move again, and the entire process is repeated. The system is found to be highly sensitive to parameters, with small parameter changes leading to large changes in overall methylation levels, and thus does not satisfy requirement C. Methylation patterns are maintained for only a few cell generations under optimal parameters and thus do not meet requirement A. Remarkably, this occurs despite freezing of the chromosome during methylation reactions. This assumption of instantaneous reactions is not consistent with requirement B.

The more recent publication^[^ ^28^ ^]^ claims to have found a “design principle of 3D epigenetic memory systems”. The proposed mechanism is essentially identical to the previous model, but the performance of the model has been dramatically improved by assuming that the number of enzymes is smaller than the number of nucleosomes, compare Figure 3B,E in Ref. [28]. The crucial element is that faithful restoration is contingent on the balance between missing marks and available methylases that deplete during the process, i.e., the mechanism requires fine‐tuning. Moreover, this model also assumes instantaneous methylation. The unphysical freezing of the chromosome after the collapse allows to “transfer” the epigenetic information in the sequence of methylated and non‐methylated nucleosomes into a frozen chromosome conformation. However, as mentioned in requirement B, experiments suggest that recovery of the missing marks is an extremely slow process: reaching the original level of methylation after duplication‐induced depletion takes about 20 h.^[^ ^13^ ^]^ On the other hand, chromosomes are highly dynamic^[^ ^30^, ^31^ ^]^ with nucleosomes diffusing in microseconds by their own diameter.^[^ ^32^ ^]^ According to the Rouse model for polymer dynamics,^[^ ^33^ ^]^ it takes about 50^2^ times longer for an array of 50 nucleosomes (the typical size of a heterochromatin block) to relax. This makes it highly unlikely that their conformations can carry any such information even on a time scale of seconds. Furthermore, chromosome conformations are completely altered when chromosomes enter the mitotic state a few hours after DNA duplication: within minutes, the distinction between eu‐ and heterochromatin compartments is lost.^[^ ^34^ ^]^ This strongly suggests that the memory of the previous epigenetic state cannot be stored inside the chromosome conformation and that we do not understand yet why epigenetic information can be robustly transferred through 50 cell generations.

**Figure 2:**
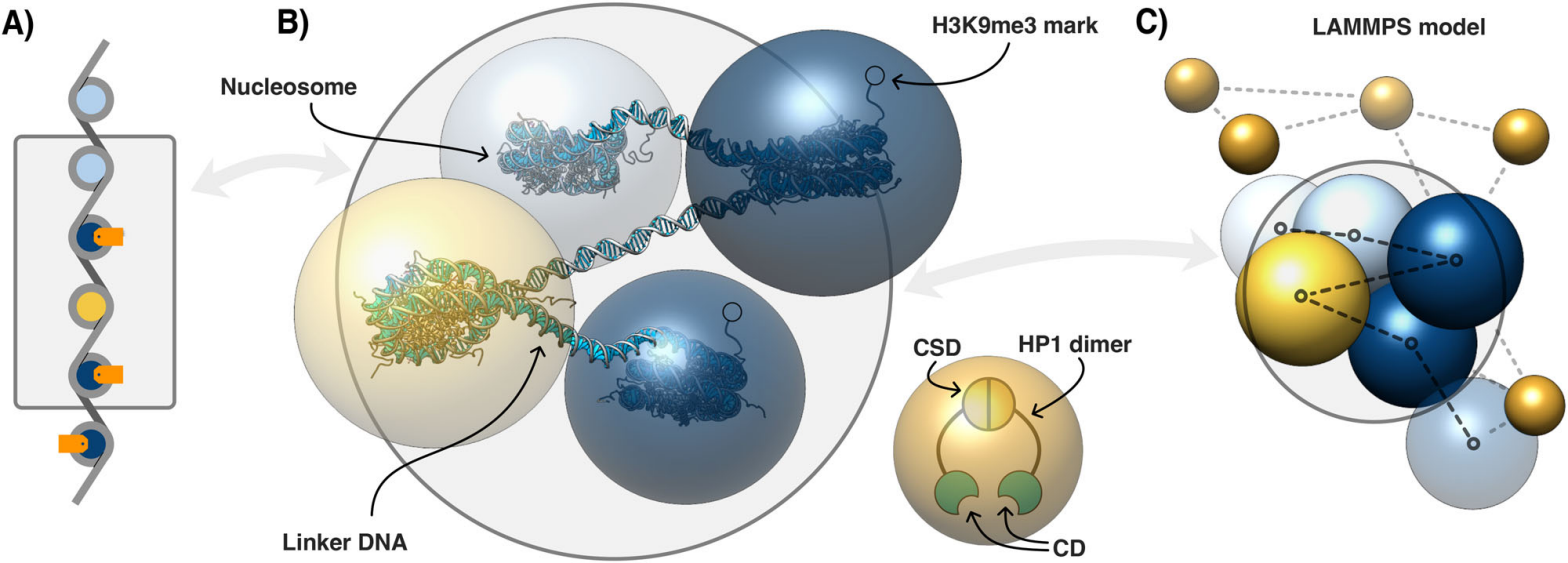
Coarse‐grained representation of chromatin and HP1s: (A) Schematic representation of chromatin section in the same style as in Figure 1. (B) Representation of the same section as in (A) in the computer model. Each nucleosome (147 bp of DNA wrapped around a histone octamer) is modelled as a hard sphere. Linker DNA (here of length 50 bp) is treated as the bond between two of these hard spheres. Two nucleosomes feature methylated marks on histone tails. Spheres are colored as follows: dark blue denotes methylated heterochromatin nucleosomes, yellow heterochromatin nucleosomes missing marks, and light blue euchromatin nucleosomes. HP1 dimers are represented by free beads (orange). HP1 molecules bind together via chromo shadow domains (CSD) to form a dimer. Chromodomains (CD) bind to methylated nucleosomes. (C) Section of chromatin surrounded by HP1 molecules as treated in LAMMPS molecular dynamics simulation. The nucleosome beads have bonded interactions (FENE) in between, which are shown by black dashed lines. HP1 beads have attractive interactions with themselves and with dark blue nucleosomes (grey dashed lines).

**Figure 3:**
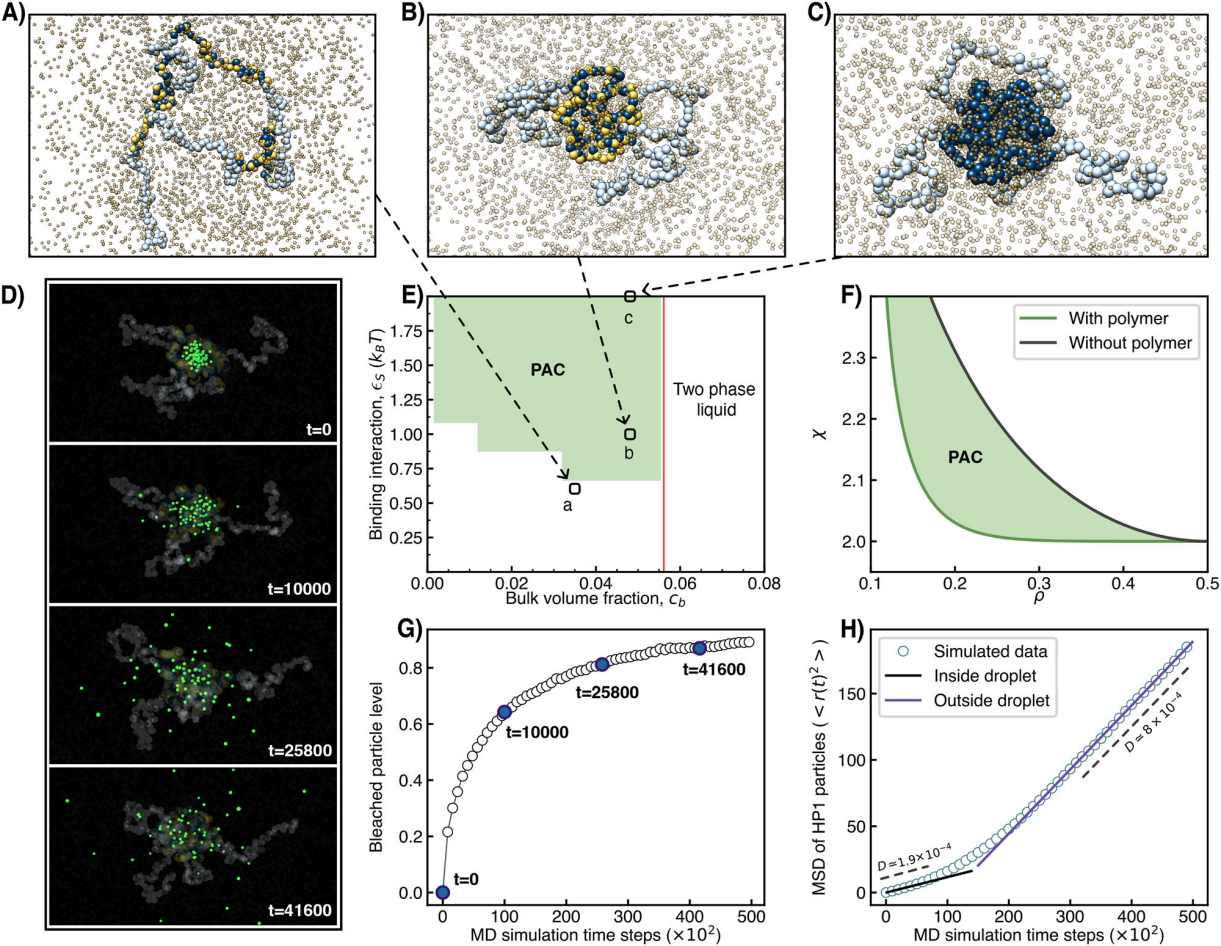
Model chromosomes induce liquid condensates: (A)–(C) Snapshots of block copolymers containing marked (dark blue) and unmarked (light blue) nucleosomes. (B) and (C) show cases that lead to condensates through PAC. In both cases, *c*_*b*_ = 0.048 and ϵ_*S*_ = 2, but for (B) one has effectively ϵ_*S*_ = 1, as every second monomer has lost its epigenetic mark (yellow monomers). (A) shows a system with a lower HP1 bulk concentration, *c*_*b*_ = 0.035, and with ϵ_*S*_ = 1.3 but with half of its markers missing and thus effectively ϵ_*S*_ = 0.65. This system is outside the PAC region, and indeed, no condensate is formed. (D) Snapshots of configurations of HP1s, at different points in time. Highlighted in green are the HPs that formed the droplet at *t* = 0. (E) Phase diagram determined from molecular dynamics simulation for χ_*S*_ = 1.1.^[^ ^35^ ^]^ Parameter values of examples (A)‐(C) are indicated for effective ϵ_*S*_‐values. (F) Phase diagram of a two‐component solution (e.g., HP1 and water) showing a miscibility gap to the right of the black curve. This gap is widened to the left (green region) in the presence of a polymer. Calculated from Equation (2) for the case of ϵ = 1 (Section S1). (G) Fraction of HPs from the bulk (at *t* = 0) inside the droplet as a function of time, averaged over 80 simulations. (H) MSD of the HP1s, which started inside the droplet at *t* = 0 (in (D) highlighted in green).

In the following, we put forward a new scheme for the restoration of epigenetic marks that does not suffer from these shortcomings, as all the information of the previous epigenetic state lies throughout the whole process in the diluted sequence of marks. Unlike in previous models,^[^ ^24^, ^28^ ^]^ where the chromosome conformational change is mainly driven by the attraction between the heterochromatic nucleosomes, in our model, the polymer only “assists” in the formation of a self‐assembled well‐defined mesoscopic reaction vessel. This feature gives the necessary robustness to a scenario where a crucial system parameter, the fraction of methylated nucleosomes, changes through the process.

Specifically, we claim that the basic underlying physical mechanism is polymer‐assisted condensation (PAC), a mechanism we discovered recently.^[^ ^35^ ^]^ In this process, molecules, here HP1, with self‐attraction are initially in the mixed state, as their concentration is too low to spontaneously demix through liquid–liquid phase separation. Addition of a polymer with attraction to the molecules, here the heterochromatin stretches of a chromosome, can induce the spontaneous formation of a droplet that contains the attractive polymer sections. The droplet stops growing once it contains all the relevant polymer sections. The result is a micellar structure with heterochromatin inside the droplet and the euchromatin stretches forming external loops. PAC‐induced condensates belong to the large class of biomolecular condensates, which have been found in the cytoplasm and inside the nucleus of cells and which fulfill various biological functions.^[^ ^36^ ^]^ PAC is one of the mechanisms that can explain how the right droplets with the right composition and size occur at the right place in the cell.

Our hypothesis is supported by in vitro experiments demonstrating HP1 to exhibit liquid–liquid phase separation at sufficiently high densities.^[^ ^37^, ^38^ ^]^ This phase separation is induced by the attraction between non‐structured regions in these proteins. In addition, as mentioned above, HP1 has a specific binding site to the H3K9me3 mark,^[^ ^6^, ^7^, ^10^ ^]^ suggesting that heterochromatin can play the role of the condensate‐inducing polymer.

The goal of this study is to demonstrate that PAC of HP1 can indeed explain the propagation of epigenetic states of cells through 50 cell generations. To achieve this, we simulate a block copolymer that represents a model chromosome under various conditions of the parameters. We chose a rather small and simple system to be able to scan the parameter space in order to demonstrate the robustness of the system in producing reaction vessels through PAC. By setting up a simple methylation scheme with a reaction speed that depends on the local HP1 concentration, we demonstrate that heterochromatin marks lost during duplication can indeed be reliably reconstructed. We propose that the well‐defined droplet surface constitutes the physical manifestation of the boundary elements, originally postulated in Refs. [6, 8, 9]. Importantly, throughout the process, the droplet retains its liquid state and the polymer shows the expected dynamic fluctuations. Interrupting the process, as it occurs, for example, during chromosome compaction and separation, followed by the initiation of an uncorrelated polymer configuration, does not hamper the restoration of the epigenetic marks.

## 2 Results

### 2.1 Reaction Container Formation Through PAC

PAC refers to the formation of a (protein) condensate due to the presence of a long flexible polymer (chromatin) in solution. In contrast to binding‐induced interactions, where the protein forms temporary bridges between monomers, in PAC only a weak attraction due to the fluctuating polymer creates a chemical potential trap for the protein inside the polymer volume, causing a condensation transition. Thus, the principal driving force to form the condensate is the tendency of demixing of the protein in bulk. As a consequence, the precondition for PAC is a protein solution that is well above the critical point in the phase diagram but undersaturated, i.e., located outside of the miscibility gap, see Figure 3F. In practical terms, this means that the bulk solution displays spontaneous phase separation if sufficiently up‐concentrated (in vitro), a scenario common to virtually all proteins involved in biomolecular condensates and, in particular, observed for HP1, the scaffold component of the heterochromatin condensate.^[^ ^37^, ^38^ ^]^ Analogous scenarios to PAC have been suggested in combined experimental‐simulation studies, particularly in relation to transcriptional condensates.^[^ ^39^, ^40^ ^]^ In the following, we briefly recall essential results from the theory of PAC; for details, the reader is referred to Ref. [35].

PAC can be understood and quantified considering a mean‐field Flory‐Huggins model^[^ ^41^ ^]^ with the free energy per volume unit and in units of *k*_*B*_ *T* in equilibrium with the bulk solution:

(1) $$\begin{array}{ccc} F(c,\phi ) & = & F_{el}\left(\phi \right)+F_{mix}\left(\phi ,c\right)+\chi c\left(1-c-\phi \right)\\ & & +\;\Pi -(\mu +\varepsilon \phi )c\; \end{array}$$

Here, *c* and ϕ denote the volume fraction of the protein and the polymer, respectively. We note that the protein concentration in contact with the polymer, *c*, differs from its bulk concentration, which we denote by ρ further below. The first two terms represent the chain elasticity and free energy of mixing of the protein and the polymer. Here, in particular, the Flory‐Huggins form is applied as $F_{mix}=\frac{1}{\nu }c\ln c+\left(1-c-\phi \right)\ln\left(1-c-\phi \right)$, where ν denotes the volume of the protein with respect to the volume of the common solvent (water). The bulk interaction parameter χ has to be chosen above the critical value (χ_*C*_ = 2 for the symmetric case ν = 1) of the pure protein‐water solution, and it is useful to define η = χ − χ_*C*_ > 0. We note that our model accounts for multivalent interactions between HP1's in a mean‐field manner. The chemical potential of the bulk, μ, has to be set to be outside of the miscibility gap so that the protein is not phase separating by itself. This means that μ < μ_0_, where μ_0_ denotes the value at coexistence. The equilibrium of the condensate with the bulk solution with respect to volume changes is given by the osmotic pressure, Π, of the proteins in the bulk. We note that −Π represents the free energy per unit volume of the bulk at given chemical potential. *F* (*c*, ϕ) is thus actually the difference between the free energy density of the condensate and the bulk. The preferential interaction ϵ of the polymer with respect to the protein is modeled in a mean‐field‐like manner. We note that if μ + ϵϕ > μ_0_, condensation is favored due to the polymer‐protein coupling. The equilibrium state is obtained by minimization of the free energy per monomer (since the number of monomers is a conserved quantity) with respect to *c* and ϕ.

While the minimization problem *F* (*c*, ϕ)/ϕ → min can only be solved numerically in the general case, in Ref. [35] we developed an analytical solution for ν = 1 using a Landau approximation of the free energy function. This allows to calculate the PAC‐transition in the form of

(2) $$\varepsilon_{PAC}={\left[\frac{\left|\mu \right|}{\delta_{0}\left(1-\delta_{0}\right)}\right]}^{1/2}\;$$

with $\delta_{0}^{2}=\frac{3}{2}\eta$. This defines a surface in the 3D parameter space (ρ, χ, ϵ), with $\mu =\ln\left(\frac{\rho }{1-\rho }\right)-\chi \left(2\rho -1\right)$ and μ_0_ = 0, where ρ denotes the volume fraction of the protein in the bulk. Within this approximation, we also calculate the region of the PAC‐state outside of the miscibility gap of the bulk solution, i.e., χ(ρ, ϵ) (Section S1). The result is plotted for the case of ϵ = 1 in Figure 3F. For ν > 1 the essential difference is that then μ_0_ < 0 and |μ| has to be replaced by |Δμ| = |μ − μ_0_|. We note that the exact location of the PAC‐transition has to be calculated numerically or, in case of simulation, by scanning the parameter space.^[^ ^35^ ^]^

A surprising result of the theory regards the volume of the condensate, which up to a prefactor of order unity is given by

(3) $$V_{PAC}≃N\frac{{\left|\mu \right|}^{-1/2}}{{\left(\delta_{0}\left(1-\delta_{0}\right)\right)}^{1/2}}\;$$

with *N* denoting the degree of polymerization of the polymer. What is particularly noteworthy is that the condensate volume is independent of the strength of the nucleosome‐protein interaction, ϵ. This is due to the fact, that the droplet is dominated by the condensed protein which saturates the polymer, i.e., the monomers are surrounded by proteins in the PAC state. This means that a stronger protein‐monomer interaction does not have much impact on the net interaction inside the droplet once the condensate is formed. Indeed, this prediction is confirmed in the simulations.^[^ ^35^ ^]^ This aspect is important in the context of epigenetic restoration because after replication, only half of the epigenetic markers are left on a given daughter chromosome and therefore the effective value of ϵ is now ϵ/2. But as long as ϵ/2 > ϵ_*PAC*_ we still have the container for the necessary enzymatic reactions and, to a good approximation, this container maintains its volume throughout the whole re‐methylation process. We believe that the robustness of the PAC transition with respect to the effective interaction offers a strong rationale for considering it as a mechanism in heterochromatin.

Here we carry out molecular dynamics simulations to study the formation of an HP1 condensate through PAC with a block copolymer representing a model chromosome (Section S2). In short, the chromosome is represented by a bead‐spring model, where each bead corresponds to a single nucleosome, and HP1 molecules are modeled by unconnected beads (of the same diameter as the monomers). We distinguish between methylated and unmethylated nucleosomes by including an attractive potential to HP1 molecules for the former. A depiction of this representation is shown in **Figure** **2**B. Methylated nucleosomes are indicated in dark blue, and unmethylated hetero‐ and euchromatin are discriminated by colors yellow and light blue, respectively. Within the simulation, there is no difference between the latter two; the color distinction purely serves to highlight the missing information. The interactions in our model involve attractive LJ potentials between the HP1‐beads of strength χ_*S*_, and between the heterochromatically marked monomers and HP1 of strength ϵ_*S*_, see Figure 2C; the index “S” indicates the values used in the simulation model. The LJ attraction between HP1‐beads mimic multivalent interactions. Interactions between monomers (regardless of whether they are marked or not) and between unmarked monomers and HP1 are modeled by purely repulsive potentials. All lengths are measured in units of the bead diameter and energies in units of *k*_*B*_ *T*. Throughout our simulations we use χ_*S*_ = 1.1, a value well above the critical point χ_*X*_ ≃ 0.9.^[^ ^42^ ^]^ In Ref. [35], we have simulated PAC for a homopolymer with a chain length of 300 monomers. The phase diagram determined in that study is shown in **Figure** **3**E, displaying the plane ϵ_*S*_ versus *c*_*b*_ (the bulk volume fraction of HP1) for fixed χ_*S*_ = 1.1. The red vertical line indicates the transition to the condensed phase which occurs at volume fraction *c*_*b*_ ≃ 0.0575.

In the current study, we use a model chromosome consisting of seven blocks. Four blocks, including the outer blocks, represent euchromatin, and three blocks are heterochromatin. Each block is 50 monomers long, which corresponds to the median length of blocks with H3K9me3 nucleosomes in humans.^[^ ^9^ ^]^ In the future, it will also be interesting to investigate the coarse‐grained representation of chromosomes using real H3K9me3 data, but for this proof‐of‐principle, we focus on a simpler toy model. In Figure 3A–C, we show three example conformations of the model chromosome for three different parameter sets, indicated by the arrows pointing at the corresponding positions in the phase diagram, Figure 3E. The system in (C) features fully methylated blocks shown in dark blue and non‐methylated blocks in light blue. The parameters are chosen such that the system lies deep inside the PAC region, see arrow to Figure 3E. We observe that the heterochromatin stretches induce a condensate, which is surrounded by a corona of euchromatin stretches. In case (B), we have removed half of the marks from the heterochromatin blocks. The corresponding monomers are highlighted in yellow but are identical with respect to their interactions with the light blue monomers. As half of the marks are missing, the interaction strength between HP1 and the heterochromatin blocks is effectively only half as strong. The arrow to the phase diagram takes this into account and shows that the system is still inside the PAC region. As expected, we find again a micellar structure with a central condensate. The snapshot in Figure 3A shows an example where the system is outside the PAC region. We note that similar micellar structures have been put forward in Ref. [43], but here the focus was on changes in outer loops due to chromatin‐binding proteins such as RNA polymerase II.

We next study the dynamics of the condensate. In Figure 3D,G,H, we track the HP1 molecules over time. Specifically, in the snapshots of Figure 3D, we highlight HP1s which are part of the droplet at *t* = 0 in green and then show their positions after different amounts of time steps. This approach shares some similarities with FRAP (fluorescence recovery after photobleaching) experiments.^[^ ^44^ ^]^ As can be seen from the snapshots, there is a fast exchange of HP1s with the bulk so that the outline of the droplet cannot be recognized anymore for the two larger times. This can also be read off the curve in Figure 3G which shows the fraction of the “bleached” HP1s inside the droplet, i.e., the HP1s that are not part of the droplet at *t* = 0. Already at *t* = 25800, about 80% of the droplet material is composed of these bleached HP1s. To further quantify the dynamics, we show in Figure 3H the mean‐squared displacement (MSD) of the marked beads as a function of time. Around *t* = 15000 the data show a crossover from a slow diffusion regime with a diffusion constant of *D* ≈ 0.00019 to a fast diffusion regime with *D* ≈ 0.00080. This crossover reflects the escape of the marked HPs from the droplet to the bulk. It is important to note that the HP1 dynamics inside the droplet is quite high since the diffusion constant is only about four times smaller than in the bulk. Moreover, also the polymer is highly dynamic, including the heterochromatin sections inside the condensate, which remain mobile, showing Rouse dynamics throughout the entire cycle of mark‐restoration (Section S3).

Overall, a dynamic picture emerges of a condensate that rapidly exchanges proteins with the surroundings and that contains highly fluctuating polymer sections. This dynamics creates opportunities and challenges. On one hand, it allows other factors like methylases to access the heterochromatin sections. On the other hand, the system cannot rely on a frozen polymer configuration to remember and reconstruct the previous epigenetic state. Rather, the epigenetic block sequence is mapped into a spatial structure via PAC, which leads to the methylation of only the “right” nucleosomes. The droplet structure delineating the methylation boundary is fully emergent, robust to structural fluctuations, and even restores after full dissociation of the co‐condensate, e.g., during mitosis. Results from computer simulations presented in the next sections demonstrate that, despite the periodic dilution of marks, this highly dynamic system can remember its original epigenetic state even after 50 cell generations.

### 2.2 Restoration of Epigenetic Marks in One Cell Generation

In this section, we demonstrate that our system allows for the restoration of missing epigenetic marks within a single cell generation and in the next section, we extend this analysis to 50 cell generations. These simulations are meant as a proof of concept, performed for one rather arbitrarily chosen set of parameters. Later in this paper, we show that this scenario is robust in the sense that it works over a large range of parameters, which supports the plausibility of our approach.

**Figure 4:**
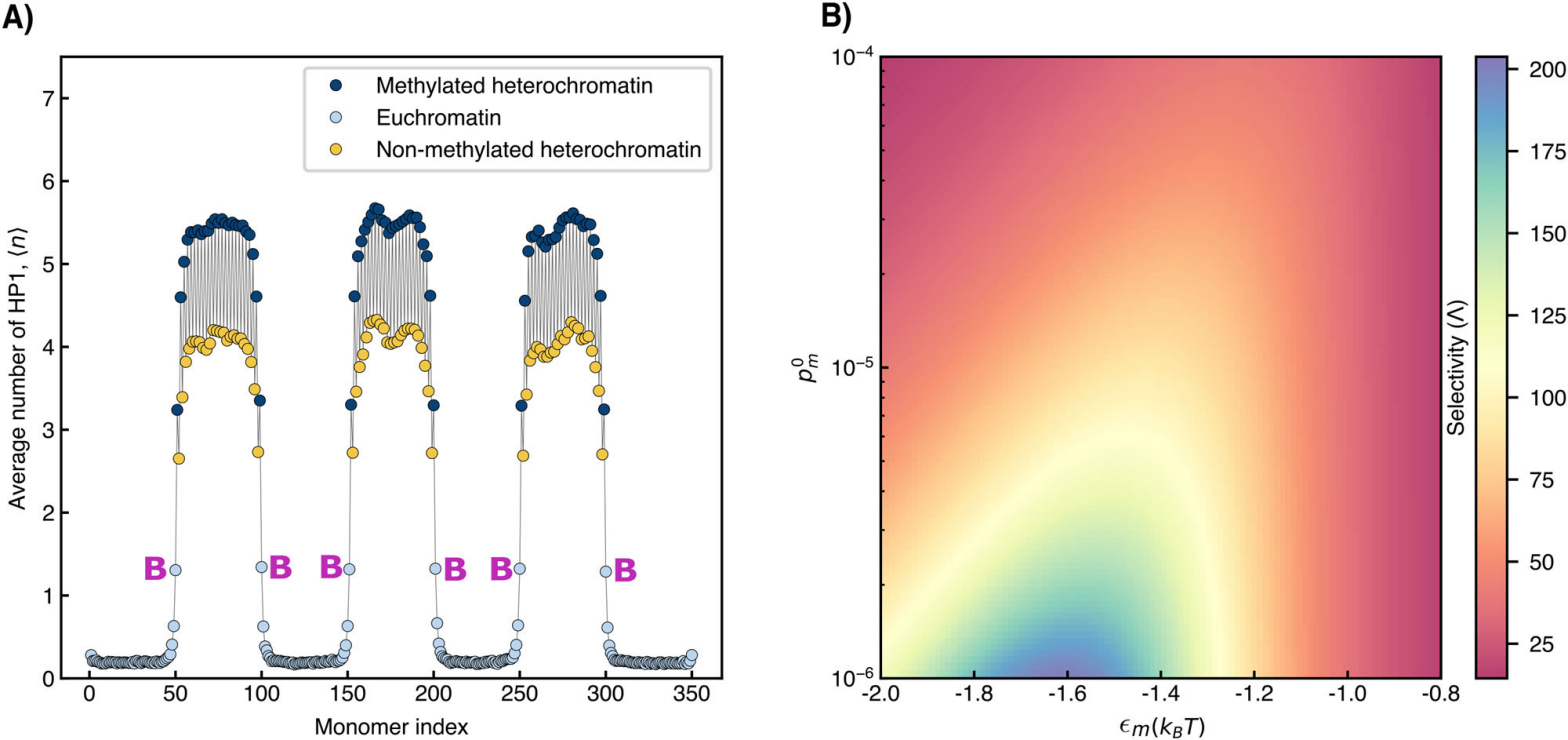
Average HP1 count and methylation probability: (A) Average number of HP1 molecules in contact with monomers of model chromosome. Heterochromatin has alternating patterns of retained (dark blue circles) and missing marks (yellow circles). The average is taken over 2000 equilibrium ensembles. Euchromatic nucleosomes next to heterochromatin domains are labelled by the letter “B”. (B) Map of selectivity Λ in the $p_{m}^{0}$–ϵ_*m*_ parameter space. The resolution of the map is 100 × 100 and each point is calculated independently. See text for details.

Our starting point is a chromosome micelle as described above with the parameters χ_*S*_ = 1.1, ϵ_*S*_ = 2, and *c*_*b*_ = 0.048, corresponding to the example shown in Figure 3B. With a chromosome micelle containing missing epigenetic marks in place, methylation reactions must be performed to restore these marks. For this mechanism to be effective, the reactions for nucleosomes lacking epigenetic marks within heterochromatin blocks must occur significantly faster than those in euchromatic blocks. The key challenge in our model arises from the fact that both types of unmarked nucleosomes are identical with respect to their interactions. Therefore, the distinction can only lie in differences of the local HP1 environment, as illustrated in **Figure** **4**A.

This plot shows the average number of HP1 molecules, 〈*n*〉, in the vicinity of given monomers, counting all HP1 within a radius of 1.5σ from these monomers, the interaction range of the LJ potential. For simplicity, we choose a sequence of epigenetic marks in which the heterochromatin blocks consist of alternating patterns of marked nucleosomes (dark blue) and unmarked nucleosomes (yellow). The HP1 number profile exhibits sharp transitions that reflect the block‐like organization of the model chromosome, with 〈*n*〉≪ 1 in euchromatin blocks and high numbers in heterochromatin blocks. In addition, the alternating sequence of marked and unmarked nucleosomes within the heterochromatin blocks leads to periodic undulations in the HP1 numbers. Importantly, the HP1 numbers of all yellow nucleosomes are higher than those of all euchromatic nucleosomes.

Note that differences in local HP1 numbers between marked (dark blue) and unmarked (light blue or yellow) nucleosomes are smallest at the boundaries between domains. Here, relative differences in numbers are only of the order two. This indicates the need for a sharp dependence of methylation rates on the local HP1 environment. Here, we assume an exponential relationship between the rate and the local HP1 number. Consequently, the probability of a randomly selected nucleosome (if unmethylated) undergoing methylation is expressed as:

(4) $$p_{m}=1-\exp\left\{-p_{m}^{0}\exp\left(-n\varepsilon_{m}\right)\right\}$$

where $p_{m}^{0}$ is the base methylation probability of a nucleosome, related to the bulk concentration of enzymes, ϵ_*m*_ is a dimensionless parameter and *n* is the number of HP1 dimers within a radius of 1.5σ (Section S4). The specific form of Equation (4) might be interpreted as a preference of the methylase to be inside the HP1 condensate, which can be expressed as an effective attractive potential between HP1s and methylases. Moreover, various studies have highlighted the propensity of the condensate environment to promote enzymatic activity,^[^ ^45^, ^46^ ^]^ which further enhances the selectivity of reactions to take place inside the reaction container. In our model, these effects are collectively accounted for by the effective potential ϵ_*m*_.

The methylation reaction rate, Equation (4), adds two new parameters to our model, $p_{m}^{0}$ and ϵ_*m*_. These parameters need to be chosen such that the methylation rates of the yellow monomers in Figure 4A are large enough compared to the methylation rates of the light blue monomers at the boundary to heterochromatin (labelled by the letter “B”), leading to a clear separation of time scales for the two types of monomers. This way, the heterochromatin blocks are restored before the blocks start to grow into the euchromatic regions. To find promising values for $p_{m}^{0}$ and ϵ_*m*_, we introduce the selectivity Λ, the ratio of the average methylation probability of the yellow nucleosomes to the average methylation probability of the light blue nucleosomes at the boundaries. Specifically

(5) $$\Lambda =\left(\frac{1}{N_{S}}\sum_{i\in S}P_{m}^{i}\right)/\left(\frac{1}{N_{B}}\sum_{j\in B}P_{m}^{j}\right)$$

where $P_{m}^{x}$ is the total integrated methylation probability of nucleosome *x*, $P_{m}^{x}=\int dn\;w_{x}\left(n\right)p_{m}\left(n\right)$ with *p*_*m*_(*n*) given by Equation (4) and *w*_*x*_(*n*) is the histone number distribution around nucleosome *x*, determined from the MD simulation. **S** and **B** describe two sets of boundary nucleosomes: **S** contains all *N*_**S**_ nucleosomes that sit at the ends of defects inside heterochromatin whereas **B** is the set of *N*_**B**_ euchromatic nucleosomes just at the border to heterochromatin blocks. Figure S1A indicates the **S**‐monomers for defects of length one, two and three nucleosomes. The rational behind this definition of Λ is that good selectivity is achieved when the **S**‐monomers get methylated first, moving the segment boundary of defects quickly inwards, before **B**‐monomers start to get methylated. Large values of Λ therefore lead to the desired separation of time scales.

In Figure 4B, Λ is plotted in the $p_{m}^{0}-\varepsilon_{m}$ parameter space. We observe a broad range of parameters where the system is highly selective with values of Λ in the range of 20 to 200. In the following we choose ϵ_*m*_ = −1.4 and $p_{m}^{0}=10^{-5}$ as a compromise between strength of selectivity and methylation speed.

Finally, we introduce one more parameter, the distribution bias *p*_*b*_, which affects the pattern of missing marks at the beginning of the simulation. It is known experimentally that nucleosomes are distributed in a globally symmetric fashion between the daughter DNA molecules, i.e., both daughter cells inherit half of the epigenetically marked histones from the parent,^[^ ^5^ ^]^ although the process is intrinsically asymmetric, as there is a leading and a lagging strand. Molecular details of the transfer remain elusive, but there is some evidence suggesting histones to be “toggled” between leading and lagging strands in a fairly regular manner, facilitated by the fork protection complex.^[^ ^47^ ^]^ In the current study, we allow for the whole range of scenarios of the local distribution, from strictly alternating to completely random. We distribute the nucleosomes between the two DNA copies, refill the missing nucleosomes with unmarked nucleosomes, and then use only one of the two resulting sequences as an input for our simulation. Figure S2 provides a visual explanation of the dilution scheme. Specifically, to create a diluted epigenetic sequence, we transfer the first nucleosome on the bottom of the entire chromosome chain to either of two newly formed strands with probability 1/2. This results in either keeping the mark or removing it (meaning the nucleosome is transferred to the other DNA copy). We then assume the probabilities for the next nucleosome to end up on the same and other DNA copy to be 1/2 − *p*_*b*_ and 1/2 + *p*_*b*_, respectively. The range of possible *p*_*b*_‐values goes from *p*_*b*_ = 0, the completely random case, to *p*_*b*_ = 1/2, the fully alternating pattern shown in Figure 4A. The more random the distribution, the larger is the probability of larger segments of nucleosomes with missing epigenetic marks (defects), which makes it more challenging to re‐establish the marks. For our proof‐of‐concept study, we chose *p*_*b*_ = 0.4, close to the alternating case but with some defects spanning over a length of two or more nucleosomes. An analysis of the effect of *p*_*b*_ on the re‐establishment of marks is provided in the section on robustness.

We are now in the position to study the methylation dynamics for a single cell generation. First, the model chromosome, initially containing heterochromatin blocks with yellow defects of missing marks, is equilibrated in an MD run. Then starts a series of Monte Carlo (MC) sweeps, each containing two steps: a set of methylation attempts followed by an MD equilibration run (Section S2). This way, the droplet‐polymer system is given time to relax its spatial conformation to the changing epigenetic sequence. Note that the MD simulation is not meant to represent the physical process in real time but just to provide the equilibration of the system.

**Figure 5:**
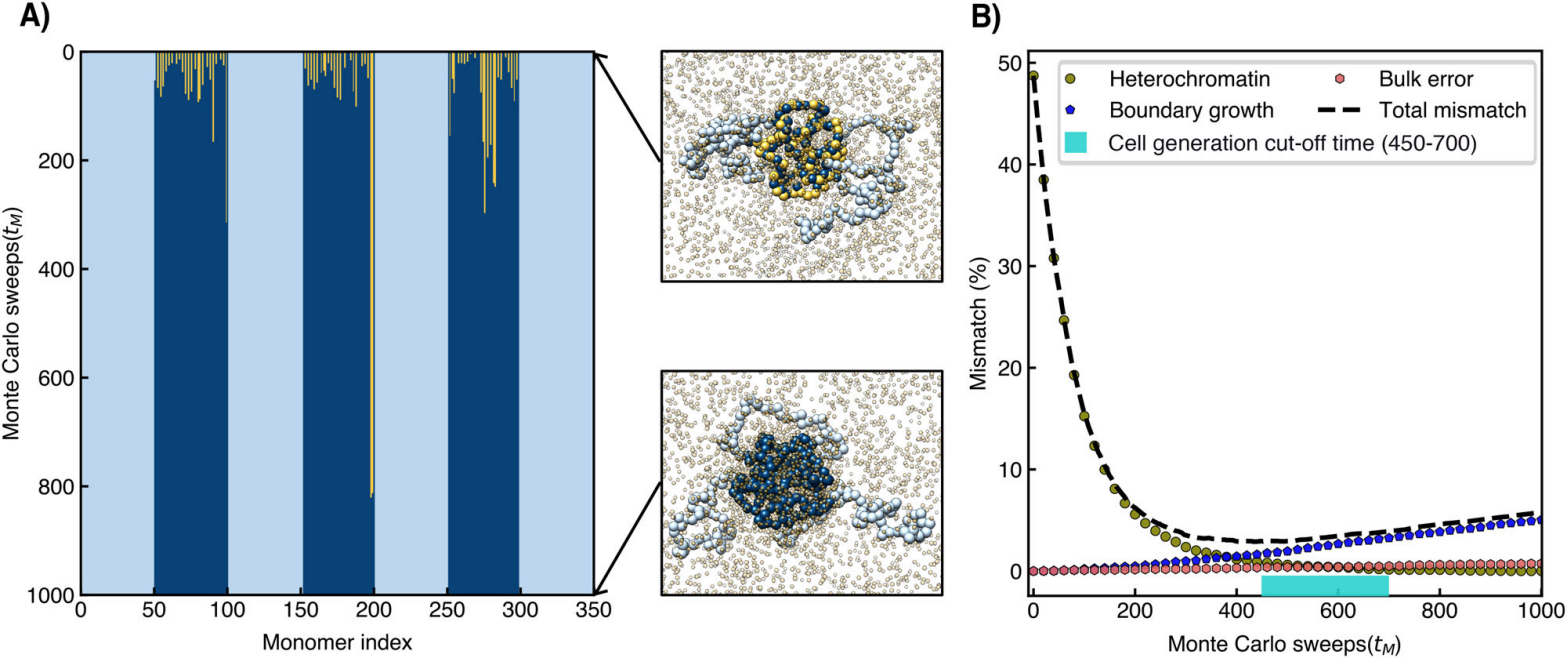
Re‐establishment of epigenetic marks in one cell generation: (A) Methylation sequence of model chromosome as a function of MC sweep number. The initial sequence was generated with a distribution bias of *p*_*b*_ = 0.4. The parameters for the methylation reaction are ϵ_*m*_ = −1.4 and $p_{m}^{0}=10^{-5}$. Representative snapshots show the system at the beginning and end of the re‐establishment run. (B) Mismatches between the current and target sequence as a function of the number of MC sweeps. The black dashed line shows the non‐monotonic behavior of the total mismatch with a minimum at around *t*_*M*_ = 400. The profiles are averaged over 100 independent simulations with different initial configurations. The cyan colored box along the time axis indicates a suitable window for choosing the cell cycle time.

**Figure** **5**A displays the evolution of the epigenetic sequence from top to bottom. We observe that with increasing time, more and more yellow nucleosomes get methylated, with most marks being re‐established after as few as 200 MC sweeps, *t*_*M*_ = 200. The last yellow nucleosome stretch, two nucleosomes long, is methylated shortly shortly after *t*_*M*_ = 800. Importantly, within the same time range, there is hardly any growth at the boundaries of the heterochromatin domains, demonstrating that our system features the required separation of time scales between methylating right and wrong nucleosomes.

This separation of time scales is further illustrated in Figure 5B, where we show the percentage of mismatch between the parent epigenetic sequence and that of the diluted sequence as a function of the MC sweeps, averaged over 100 independent simulations (Section S5). The mismatch of the heterochromatin starts at 50% and decays rapidly with the MC sweeps. Moreover, there is a slowly linearly growing mismatch in the euchromatic fraction of the chromosome, which has two contributions: the dominant boundary growth (the growth of heterochromatin into the euchromatin) and a very slow bulk error, stemming from heterochromatin that spontaneously forms within the euchromatin sections. The minimum in the total number of mismatches is reached around *t*_*M*_ = 400.

**Figure 6:**
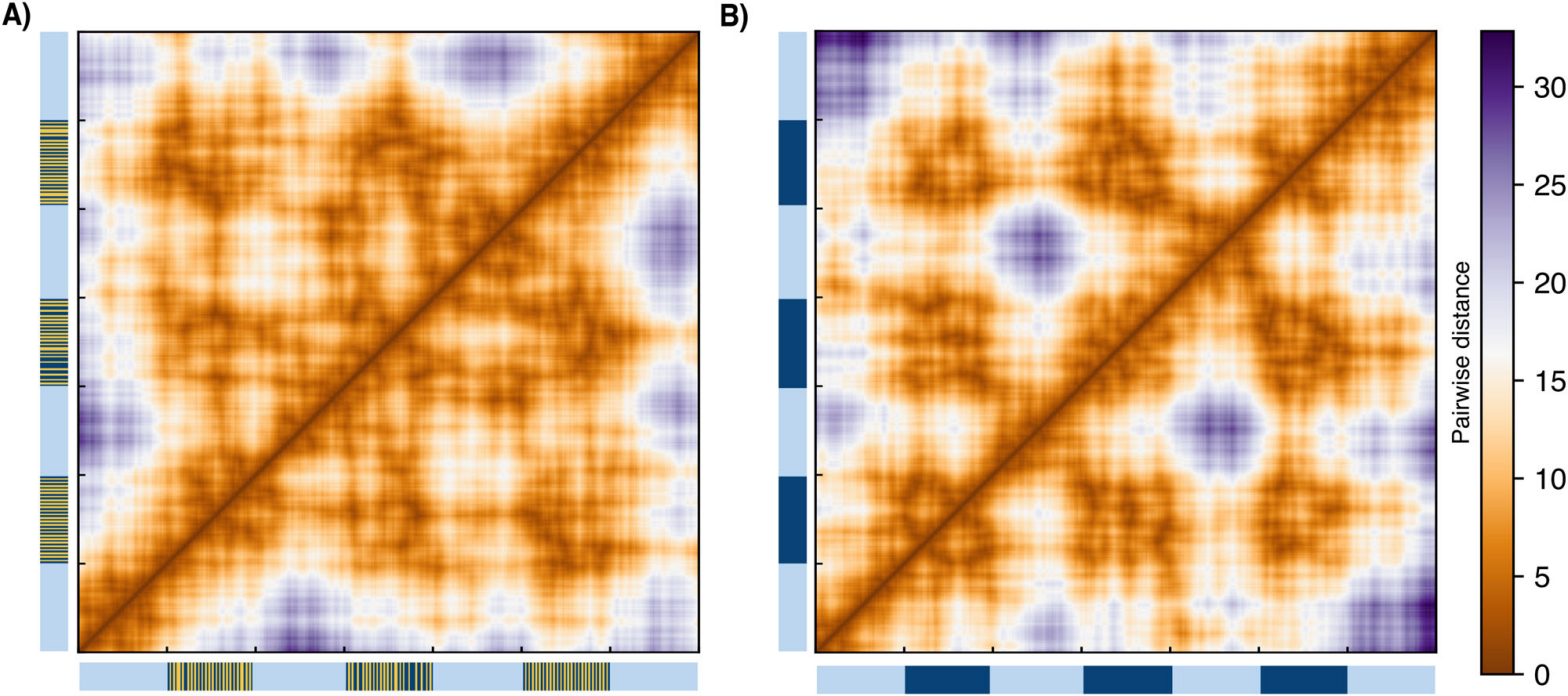
Contact maps of PAC induced micelles: Example of epigenetic restoration in one cell cycle starting from (A) a snapshot of the half‐methylated state just before methylation reactions start and (B) after *t*_*M*_ = 320 methylation sweeps, where the domains are already fully methylated. The color code corresponds to pairwise distances, calculated as $d_{ij}=\sqrt{{\left(r_{i}-r_{j}\right)}^{2}}$, where **r**_*i*_ and **r**_*j*_ are the position vectors of *i*th and *j*th monomer, respectively. The resolution of the maps is 350 × 350, i.e., one pixel per nucleosome. Methylation states are indicated in the horizontal and vertical bars next to the maps. Parameters are ϵ_*m*_ = −1.4 and $p_{m}^{0}=10^{-5}$ and *p*_*b*_ = 0.4.

Finally, **Figure** **6** shows two contact maps (or rather distance maps) for two snapshots of PAC‐induced micelles for a one cell generation simulation. Figure 6A presents the case just before methylation reactions start and (B) when full re‐methylation has occurred. Both maps show nine blocks that correspond to the three heterochromatin sections residing in the central droplet. This pattern stays intact throughout the whole cell cycle, showing the robustness of the PAC‐induced micelle.

### 2.3 Epigenetic Marks Restoration Through Many Cell Generations

We now extend our proof‐of‐concept simulation to 50 cell generations, i.e., into the range of the Hayflick limit, which, as mentioned above, represents the limited number of divisions of a normal somatic cell.^[^ ^12^ ^]^ To do so, we repeat the simulation from the previous section 50 times. At each generation, we stop the simulation after *t*_*C*_ = 600 MC sweeps, a value where the mismatch has grown slightly above its minimal value, see Figure 5B. We pick the epigenetic sequence from that last sweep as input for the next cell generation. Specifically, we remove half of the marks with a distribution bias *p*_*b*_ = 0.4 from that sequence, restart the system with a random configuration, perform an equilibration run during which a new condensate forms, and then run again for *t*_*C*_ MC sweeps.

**Figure 7:**
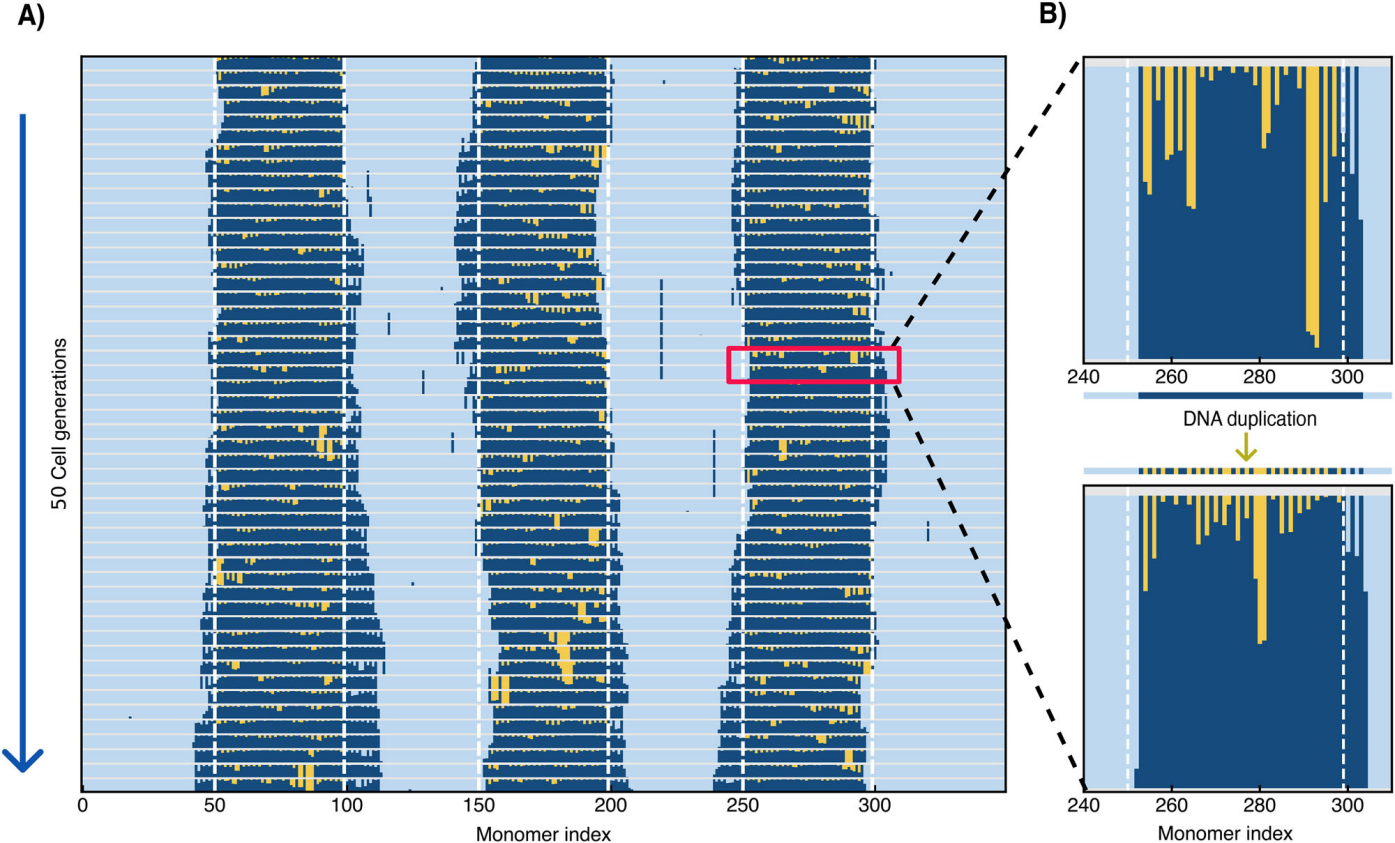
Multi‐cell generation simulations of epigenetic restoration: (A) Epigenetic mark dilution and re‐establishment through 50 cell generations. The original positions of the six euchromatin/heterochromatin boundaries are marked with dashed white vertical lines. The restoration profile shows fairly stable heterochromatin domains with some bulk error arising rarely. (B) A zoomed‐in (and vertically stretched) view of two consecutive cell generations. Between two cell generations, the sequence from the end of the previous generation is diluted by removing half of the marks (mimicking the effect of DNA duplication). The resulting sequence serves as input for the next cell generation.

The resulting time development of the epigenetic sequence is shown in **Figure** **7**. We observe that even after 50 generations, the block‐like arrangement of eu‐ and heterochromatin is still intact, including the sizes and positions of the domains. This is remarkable since each time when half of the marks are removed, a given heterochromatic domain shrinks by an average of one nucleosome. One might thus expect that the domains, at the start each being 50 nucleosomes long, disappear after 50 cell generations. However, Figure 7 lets us conclude that the shrinkage of the domains is roughly cancelled by an outward drift of heterochromatin into the euchromatic domains. As a result, the boundaries show diffusive trajectories, as can be seen by comparing the boundary positions over time with the original boundaries, indicated by white dashed lines. Our choice of the number of MC sweeps per generation, slightly above the time that minimizes the total error per generation, might play a role here.

Another noteworthy observation in Figure 7 is the emergence of a long‐lived defects, characterized by wide gaps of unmethylated heterochromatin. For instance, such a defect occurs in the middle block in the 40th cell cycle generation as a sequence of five unmarked nucleosomes and persists up the 43rd cell cycle where the defect rapidly disappears. This observation suggests that the re‐establishment mechanism is capable of “healing” larger defects, though the process may require several cell generations, as larger defects have the tendency to reside at the surface of the droplet.

We occasionally observe the spontaneous formation of isolated marks within euchromatin domains. These defects remain isolated and disappear after a few cell generations. This is a consequence of cell division‐induced mark dilution, which stabilizes euchromatin domains even in the absence of enzyme‐mediated catalytic mark removal.

### 2.4 Robustness of Restoration Scenario

So far, we have provided a proof of concept for the re‐establishment scenario for one set of parameters. In the current section, we give evidence that the scenario is rather robust with respect to changes in the parameters. We start by reiterating the point that PAC itself is already very robust. As can be seen in Figure 3E,F, there is a large range of parameters where PAC occurs. Moreover, as the free energy of the polymer‐condensate is dominated by the condensate proteins, many quantities, such as the condensate volume, Equation (3), do not depend on ϵ_*S*_, the attraction strength between the marked monomers and HP1. In fact, the map of selectivity Λ in the $p_{m}^{0}$–ϵ_*m*_ parameter space in Figure 4B (for ϵ_*S*_ = 2) shows little change as we vary this value from ϵ_*S*_ = 1.7 to 2.5 in Figure S3. The selectivity only drops dramatically once the system approaches the boundary between PAC and the mixed state, which is the case for ϵ_*S*_ = 1.5. Thus, the selectivity is closely linked to the robustness of PAC.

We also note that the re‐establishment process can be interrupted at any moment (e.g., by switching off the attraction between HP1 and the methylated nucleosomes) and then be restarted (e.g., by switching the attraction on again) without compromising the faithful re‐establishment of the epigenetic sequence. An example is provided in the Figure S4. This feature is important since the re‐establishment of the markers must be interrupted during the dramatic rearrangement of chromosomes during mitosis, in which the distinction between eu‐ and heterochromatin is temporarily disrupted.^[^ ^34^ ^]^

**Figure 8:**
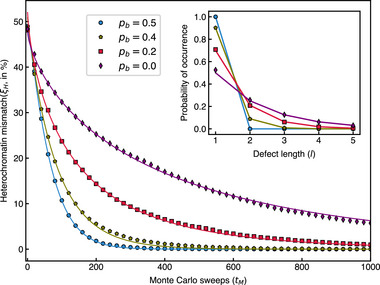
Decay of heterochromatin mismatch with time for different values of *p*_*b*_: The plot shows that the re‐establishment speed varies strongly with *p*_*b*_. Each plot is obtained by averaging over 100 simulations. Solid lines were obtained by fitting Equation (6) to the data. Inset: Probability distributions of defect sizes for different values of *p*_*b*_. The data points are obtained from simulating the dilution process on the chromosome model whereas the solid lines represent the theoretical estimate for the probability (1/2 − *p*_*b*_)^*l* − 1^(1/2 + *p*_*b*_) for a defect of length *l* to occur (at the start of a defect, the defect continues for exactly *l* − 1 additional nucleosomes with probability (1/2 − *p*_*b*_)^*l* − 1^(1/2 + *p*_*b*_)); this estimate works well for defect lengths *l* that are much shorter than the length of a heterochromatin domain.

We focus next on the robustness of the scenario on parameters related to mark dilution and reaction speed. In the proof‐of‐concept simulation, we assumed that nucleosomes are distributed between the two DNA copies with a distribution bias of *p*_*b*_ = 0.4. The bias affects the distribution of defects inside the heterochromatin, as shown in the inset of **Figure** **8**, which depicts the probability of occurrence of different‐sized defects for various values of *p*_*b*_. While *p*_*b*_ = 0.5 produces an alternating pattern where all defects consist of a single nucleosome, reducing *p*_*b*_ to 0.4 results in defects of length two in approximately 10% of cases.

As suggested before by Figure 7, defects spanning multiple marks may require several cell generations to be repaired. This can be clearly seen when inspecting the selectivity for chromosomes with alternating blocks of marked and unmarked nucleosomes of different lengths. While single‐block defects exhibit high selectivity (see Figure 4B), larger blocks show significantly reduced selectivity, as shown in Figure S1D,E. This raises the question of whether, in scenarios with greater randomness than *p*_*b*_ = 0.4, our model can still successfully re‐methylate heterochromatin before epigenetic marks encroach into the euchromatin domains. To address this, we examine the evolution of the heterochromatin mismatch ξ_*H*_ (defined in Section S5) as a function of the MC sweep, shown in Figure 8. As evident from the plot, the decay of the heterochromatin mismatch slows significantly with decreasing distribution bias *p*_*b*_. Notably, these curves deviate from simple exponential behavior but are well approximated by compressed or stretched exponentials:

(6) $$\xi_{H}\left(t_{M}\right)={\xi_{H}}^{0}\exp\left(-{\left(t_{M}/\tau \right)}^{\nu }\right)$$

where the exponent ν depends on *p*_*b*_. In the alternating case (*p*_*b*_ = 0.5), the exponent ν is slightly greater than one (ν ≈ 1.14), suggesting that the local density around non‐methylated nucleosomes increases during the mark re‐establishment process, a second‐order effect in the PAC scenario. For all other simulated cases, the fitted parameters are given in Section S6. This reflects the fact that epigenetic sequences of heterogeneous defect sizes are characterized by locally different methylation probabilities, i.e., larger defects require more time for re‐methylation.

**Figure 9:**
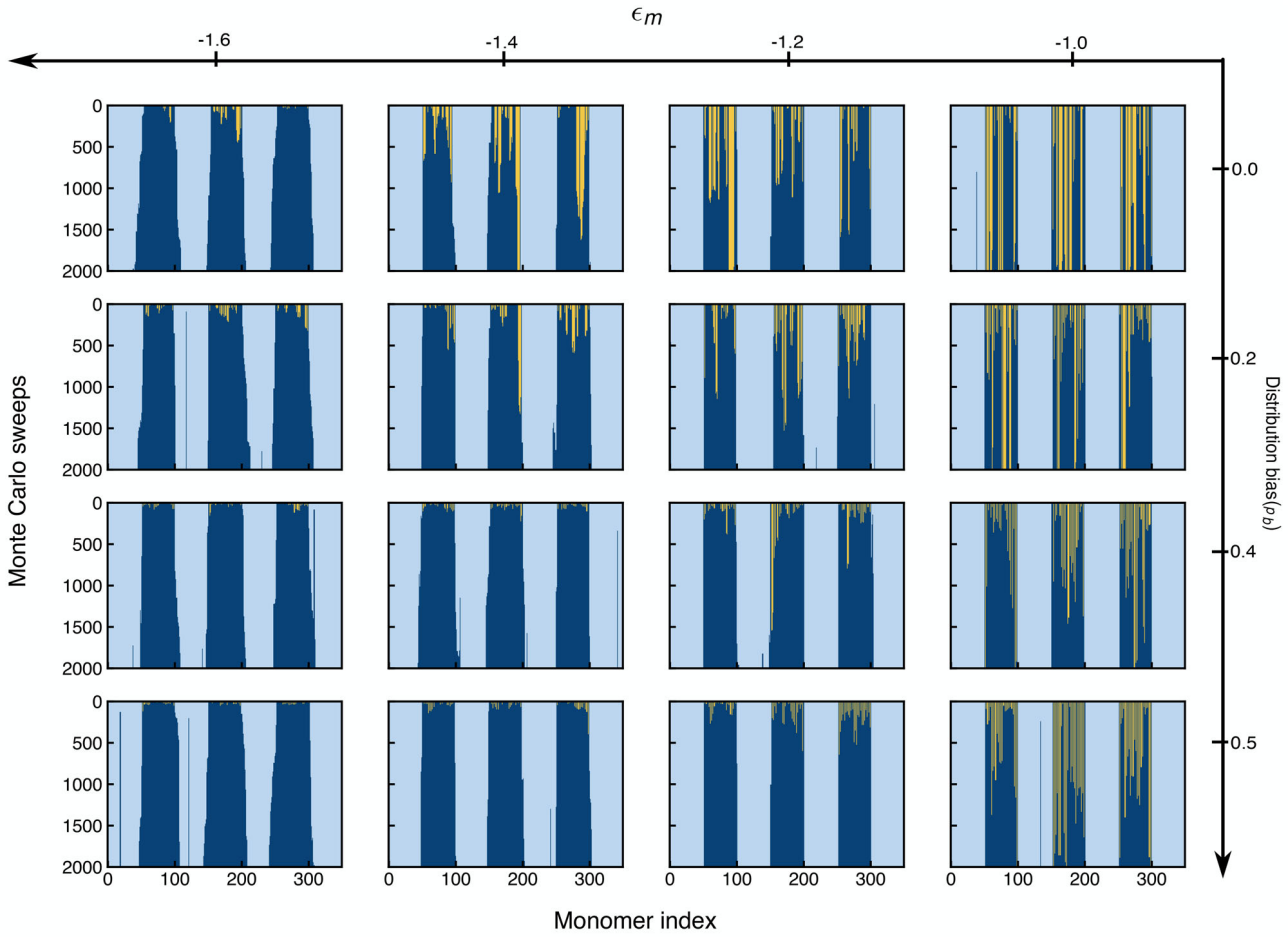
Single cell cycle profiles across a vast parameter landscape: Evolution of methylation status of nucleosomes through a cell cycle for different values of ϵ_*m*_ from Equation (4) and of the distribution bias, *p*_*b*_. As can be seen from the various plots, the restoration mechanism works across the entire range of parameters displayed, yet the time scales for restoration differ significantly.

Next, we test whether our scenario is still able to repair the defects before heterochromatin spreads into euchromatin, despite the much longer recovery timescales for more random sequences. To do this, we study in **Figure** **9** the evolution of epigenetic sequences for a single cell generation for a long period of 2000 MC sweeps for the *p*_*b*_‐values discussed above. In addition, we also vary the value of ϵ_*m*_ from Equation (4), namely ϵ_*m*_ = −1.0, −1.2, −1.4 (the previously used value), and −1.6. As can be seen from the various plots, the defect closing occurs faster for larger *p*_*b*_‐values, i.e., smaller defect sizes, and more negative ϵ_*m*_‐values. This reflects the fact that nucleosomes inside smaller defects will be surrounded by more HP1 molecules and methylation rates increase exponentially with ϵ_*m*_. Notably, however, within the entire range of parameter values considered in Figure 9, the time scale for closing the defects is faster than the time scale for growing into euchromatin, suggesting that the scenario is robust everywhere.

Finally, we discuss the robustness of the scenario with respect to the cell cycle time. In the Figure S5, we show various examples of time developments of epigenetic sequences for 50 cell generations. In all examples, we use again the parameters from our proof‐of‐concept study with the exception of the cell cycle time (counted by the number of MC sweeps). Specifically, we consider *t*_*C*_ = 450, 500, 550, 600 (the value used in Figure 7), 650 and 700. For the shortest cell cycle time, we observe a slight inward drift of the heterochromatin boundaries and for the longest, an outward drift. Nevertheless, for the whole range of values considered here, the starting sequence can still be recognized after 50 cell generations.

In some cases the pattern changed substantially. A case where one heterochromatin domain almost disappeared is shown in Figure S6A for *t*_*C*_ = 450. A case where a new domain formed between two domains, grew, eventually connecting these domains is displayed in Figure S6B for *t*_*C*_ = 750. To gain a better understanding of the typical behavior, we introduce the average restoration score 〈ρ_*R*_〉 which is a function of the monomer index *i* (Section S7). Figure S7 provides the score for four different *t*_*C*_‐values, ranging from *t*_*C*_ = 500 to 650. The plots show that with increasing *t*_*C*_, the heterochromatin domains show increasingly perfect restoration scores (〈ρ_*R*_〉 = 1), indicating successful maintenance of the epigenetic marks across 50 consecutive cell generations, for each simulation. However, heterochromatin starts to invade euchromatin domains, leading to profiles with negative restoration scores, 〈ρ_*R*_〉 < 1, near the ends of the euchromatin domains, indicating incorrect methylation events. Nevertheless, Figure S7 demonstrates that the overall restoration works remarkably well in the range of cell cycle cut‐off times depicted. This can also be seen by inspecting the cumulative restoration score for heterochromatin domains in Figure S8. To improve the performance even further, we would need to introduce two missing elements in our scenario: one that makes the re‐establishment of epigenetic marks independent of cell cycle time, and one that suppresses the spontaneous formation of new heterochromatin domains. We further discuss the possible nature of these missing elements at the end of the discussion section.

### 2.5 Discussion

We put forward a novel scenario for the re‐establishment of the epigenetic state of cells after DNA duplication. Key in this scenario is the formation of a liquid reaction container by polymer‐assisted condensation (PAC) where the chromosome sections responsible for the condensate formation are simultaneously the sections that need to be restored. With only these sections residing inside the reaction container, enzymes in the condensate add marks mostly to nucleosomes at genomic positions that had been epigenetically marked in the previous cell generation. Using computer simulations, we demonstrated that this self‐organised system is indeed capable of re‐establishing the epigenetic state 50 times in a row, each time starting epigenetically diluted by a factor of two. This is consistent with the number of cell generations given by the Hayflick limit.^[^ ^12^ ^]^

The proposed scenario exhibits remarkable robustness to external perturbations. Both the changes of parameters and the complete interruption and subsequent restart of the process have little effect on the restoration of the epigenetic marks. Robustness is a general characteristic of living systems. However, the level of robustness required for this process to function effectively is particularly stringent: (i) During DNA duplication, half of the epigenetic marks are lost, which effectively reduces the interaction strength between the heterochromatic sections and HP1 by a factor of two. PAC theory^[^ ^35^ ^]^ shows that the condensate has the remarkable property to be independent of this interaction parameter (see also Equation (3)). (ii) Experiments based on quantitative mass spectroscopy suggest that the restoration of histone lysine methylation is an extremely slow process, taking about 20 h.^[^ ^13^ ^]^ This process is interrupted by mitosis, where the interphase chromosome structure is lost within minutes.^[^ ^34^ ^]^ After cell division, each chromosome needs to refold to an interphase state which allows the epigenetic restoration to continue. In fact, as demonstrated in Figure S4, our simulations can be stopped at any time and restarted with a new initial configuration. PAC will then automatically self‐organise the system into a structure that continues the process of epigenetic re‐establishment.

The other computational studies^[^ ^24^, ^28^ ^]^ for the restoration of epigenetic states do not have these properties. In contrast to our study, the heterochromatic monomers either attract each other either directly^[^ ^28^ ^]^ or through a bridging‐type interaction.^[^ ^24^ ^]^ In both cases, the conformational dynamics of the polymer is stopped before epigenetic marks are removed and during their restoration. The methylation reactions are performed on a frozen structure, with denser regions being methylated. The outcome of the reaction scheme is independent of the initial epigenetic sequence, and the reactions reconstruct the marks even if the marks were completely removed.^[^ ^24^ ^]^ The epigenetic restoration scenario in Ref. [24] is not robust to parameter changes, see also Ref. [28]. Limiting the number of available enzymes in Ref. [28] improves performance, but the size of the heterochromatic domains now directly reflects the number of available enzymes. In contrast, our scenario is robust to parameter changes and allows for dynamic rearrangement of chromatin during the remethylation process. Our model shifts the focus from the role of specific conformational properties of chromatin to the collective behavior due to the formation of a biomolecular condensate.

To conclude, we have proposed the first physically plausible scenario for cellular memory: epigenetic restoration based on PAC. Crucial elements of the scenario include: (1) a liquid reaction vessel as the only mesoscopic stable structure in this process, (2) a sharp boundary of the condensate constituting the physical realization of the elusive boundary elements,^[^ ^6^ ^]^ and (3) enzymatic reactions with a preference for the condensate. Our scenario leads to a possibly unique combination of two types of robustness: independence of droplet properties from the polymer‐protein interaction strength and independence from the initial chromosome conformation, allowing the process to be interrupted, as it likely occurs when mitosis interrupts the re‐establishment process.

A possible weakness of our scenario is the requirement for cell cycle times to be chosen in a relatively small range to avoid systematic drift of heterochromatin domain boundaries. Small cycle times lead to a high probability of domain shrinkage, while large cycle times lead to likely domain expansion. Closely related to this, our scenario does not suppress the spontaneous formation of a new heterochromatin domain within euchromatin. We believe that both problems can be resolved at the same time by introducing a more complex reaction scheme. In our current study, we simplified this system by assuming that there is only one reaction, the (full) methylation of a nucleosome. However, it is known that the H3K9me3 mark is produced in three separate steps.^[^ ^1^ ^]^ Moreover, there is also a demethylation enzyme present.^[^ ^1^ ^]^ This suggests that the accuracy of this process might be improved via kinetic proofreading,^[^ ^48^ ^]^ where the methylation of a “wrong” nucleosome can be corrected. Also, the presence of both methylase and demethylase, with orthogonal selectivity with respect to the heterochromatin condensate, allows for a scenario where the activity of both enzymes cancel each other out at heterochromatin boundaries. This might make our process independent of the cell cycle time. We plan to implement these additional features into our model in a future study.

## Author Contributions

J.‐U.S. and H.S. conceived, designed, and supervized the research. S.M. and E.S. developed the software; S.M. wrote the analysis code. H.M. provided computational tools. S.M. performed all simulations and data analysis. All contributed to data interpretation and research implementation. S.M., J.‐U.S., and H.S. wrote the manuscript; all authors discussed results and approved the final version. For the revision, S.M. performed additional simulations, theoretical work, and, with H.S., re‐analyzed data and drafted reviewer responses. J.‐U.S. addressed specific reviewer points. H.S. and S.M. revised the manuscript, with E.S. assisting with writing and tooling.

## Conflicts of Interest

The authors declare are no conflicts of interest.

## Conflict of Interest

The authors declare no conflict of interest.

## Supporting information



Supporting Information

## Data Availability

The data that support the findings of this study are available from the corresponding author upon reasonable request.
