# Supplementary material for: A Self‐Organized Liquid Reaction Container for Cellular Memory
Source: Adv Sci (Weinh). 2026 Jan 12;13(20):e12500. doi: 10.1002/advs.202512500 (PMC13067766; doi:10.1002/advs.202512500)
Supplement: Supplementary file 1 — Supporting Information [file ADVS-13-e12500-s001.pdf]

Supporting Information for

A self-organised liquid reaction container for cellular memory

Sukanta Mukherjee, Enrico Skoruppa, Holger Melitz,

Jens-Uwe Sommer*, Helmut Schiessel*

* sommer@ipfdd.de , * helmut.schiessel@tu-dresden.de

S1 Derivation of PAC region

Here we describe how the two lines in Fig. 3(F) were obtained. The bulk chemical potential is given by

$$\mu = \ln\left(\frac{\rho}{1-\rho}\right) + \chi(1-2\rho). \tag{S1}$$

The symmetry of the bulk solution is broken by μ only. Thus the coexistence line follows from $\mu = 0$ which leads together with Eq. S1 to

$$X_x(\rho) = \frac{\ln\left(\frac{\rho}{1-\rho}\right)}{2\rho - 1}. \tag{S2}$$

This equation was used to draw the inner line in Fig. 3(F).

To calculate the outer boundary of the PAC region we start from Eq. 2:

$$\mu\delta_0(1-\delta_0) = -\epsilon^2. \tag{S3}$$

which follows from the Landau expansion. Inserting Eq. S1 into Eq. S3 and using the definition

$$\delta_0^2 = \frac{3}{2}(\chi - 2) \tag{S4}$$

we arrive at

$$\frac{\ln\left(\frac{\rho}{1-\rho}\right) + \left(\frac{2}{3}\delta_0^2 + 2\right)(1-2\rho)}{\delta_0(1-\delta_0)} = -\epsilon^2. \tag{S5}$$

Introducing $a = \frac{2}{3}(1-2\rho)$ we rewrite Eq. S5 as

$$\left(a - \epsilon^2\right)\delta_0^2 + \epsilon^2\delta_0 + b = 0 \tag{S6}$$

where we introduced $b = \ln\left(\frac{\rho}{1-\rho}\right) + 3a$. One can check that $b < 0$ for $\rho < 1/2$ and that for $\rho = 1/2$ both $a = 0$ and $b = 0$. Solving the equation for δ_0 we obtain

$$\delta_0 = \frac{\epsilon^2}{2(\epsilon^2 - a)}\left(1 \pm \sqrt{1 + \frac{4b(\epsilon^2 - a)}{\epsilon^4}}\right). \tag{S7}$$

We consider in the following the solution with the minus sign as it leads to $\delta_0 \to 0$ for $\rho \to 1/2$. Using Eq. S4 we finally arrive at

$$\chi(\rho, \epsilon) = \frac{2}{3}\left[\frac{\epsilon^2}{2(\epsilon^2 - a)}\left(1 - \sqrt{1 + \frac{4b(\epsilon^2 - a)}{\epsilon^4}}\right)\right]^2 + 2. \tag{S8}$$

This equation was used to plot the outer curve of the PAC region in Fig. 3(F).

S2 Computational model

We carry out molecular dynamics (MD) simulations using a standard bead-spring model for the polymer, which represents the model chromosome. Polymer connectivity is modelled via Finite Extensible Nonlinear Elastic (FENE) potentials. We use the *Kremer-Grest* parameters [1] in our simulations, i.e., $K = 30, r_0 = 1.5\sigma, \sigma = \epsilon = 1$. We model HP1 molecules as free beads of the same diameter as the monomers in the background of an implicit solvent model. Pair interactions are Lennard-Jones potentials

$$U_{\rm LJ}(r) = 4\epsilon_{\rm LJ}\left[\left(\frac{b}{r}\right)^{12} - \left(\frac{b}{r}\right)^{6} - \left(\frac{b}{r_c}\right)^{12} + \left(\frac{b}{r_c}\right)^{6}\right] \tag{S9}$$

where $b = 1$ is the bead diameter (in LJ units), $\epsilon_{\rm LJ}$ is the interaction strength and r_c is the cutoff distance. Interactions between monomers are truncated at $r_c = 2^{1/6}$ and are thus purely repulsive; we use $\epsilon_{\rm LJ} = 1$ throughout. The same holds for the interaction between HP1 and unmarked monomers. There are attractive interactions between the HP1-beads, characterized by $\epsilon_{\rm LJ} \equiv \chi_S$, and between the epigenetically marked monomers and HP1, given by $\epsilon_{\rm LJ} \equiv \epsilon_S$; both interactions are truncated at a cut-off distance of $r_c = 2.5$. In the absence of HP1, the polymer is thus in a good implicit solvent. We note that due to the hard-core repulsion, a minimum value of ϵ_S, $\epsilon_{S0} \simeq 0.6$, is necessary to realize a crossover from repulsion to adsorption of the HP1-beads with respect to the polymer chain [2]. The bulk phase diagram of the LJ-system has been studied before [3] and the critical point

was found to be located at $\chi_X \simeq 0.9$ and $c_X = 0.32$. Throughout our simulations we use $\chi_S = 1.1$, which is well above the critical point. The transition to the condensed phase is then located at an HP1 bulk concentration of about $c_b \simeq 0.075$. Simulations are carried out using the LAMMPS MD package [4] in a cubic box of length $L = 50$ using periodic boundary conditions.

The model chromosome is a block copolymer consisting of 350 monomers (= nucleosomes). We intialize simulations with an epigenetic sequence consisting of seven alternating blocks, four of which are unmarked (euchromatin blocks) and three are epigenetically marked (heterochromatin blocks). At the beginning of each cell generation, half of the marks are removed, as specified in more detail below. We then let the system equilibrate via an MD run. Equilibration is assessed by monitoring the radius of gyration of the heterochromatin domains which initially collapses due to PAC.

We then carry out a series of MC sweeps in which we attempt to methylate nucleosomes, followed by MD runs to equilibrate the system. The number of attempts per sweep is always the same and is set to the total number of nucleosomes with missing epigenetic marks at the beginning of the cell cycle, which is 75. Nucleosomes are randomly picked independent from their methylation state and, if unmethylated, are then methylated with the probability p_m, given in Eq. 4 of the main text. After finishing an MC sweep, we allow the system to respond to the changed sequence by performing an equilibration run of 50000 MD timesteps. As can be seen from Fig. 3(D), the droplet has exchanged most of its HP1 molecules by then. Furthermore, after this time, the monomers already show classical subdiffusion (see Fig. S9, Supporting Information, and the section on polymer dynamics below), indicating that the polymer is at least locally in equilibrium.

S3 Polymer dynamics inside droplet

Here we study the dynamics of the heterochromatin sections. Unlike in earlier studies [5, 6], our polymer is free to move during the re-establishment of the epigenetic state and is highly dynamic. We show this here by reporting the dynamics for both extremes, the fully and the half-methylated states, using two different methods. In the first approach, we report the mean squared displacement

(MSD) of the heterochromatin sections with respect to the center of mass of the droplet:

$$< {r_d}^2 >= \frac{1}{N_M} \sum_{i=1}^{N_M} \{(\vec{\Gamma}_i(t) - \vec{\Gamma}_{CM}(t)) - (\vec{\Gamma}_i(0) - \vec{\Gamma}_{CM}(0))\}^2 \tag{S10}$$

where the summation is over all N_M monomers that belong to heterochromatin sections. $\vec{\Gamma}_i(t)$ is the position of monomer i and $\vec{\Gamma}_{CM}(t)$ is the center of mass of the condensate, both at time t. In Fig. S9 (Supporting Information) the MSD is plotted for fully methylated and half-methylated heterochromatin. Both curves show subdiffusive behaviour with an exponent 1/2, compatible with Rouse dynamics, see Eq. (5.126) in Ref. [7]. The Rouse regime sets in earlier for the half-methylated polymer which, for any given time, also shows overall larger MSD values. For longer times, not shown here, the MSD converges to a finite value, set by the size of the condensate. At $t = 50000$, the time between two MC sweeps in our cell cycle simulations, the polymer already shows subdiffusive dynamics, indicating that the monomers are locally in equilibrium at this time.

To gain a better insight into the dynamic conformational rearrangement of the polymer sections inside the condensate, we introduce the neighbourhood-neighbourhood correlation function C_{NN} which tracks the changes in the neighbourhood of monomers by mapping each monomer's proximity inside the droplet to a binary state. Specifically

$$C_{NN}(\Delta t) = 1/({N_M}^2 - N_M) \sum_i \sum_j n_{ij}(t_0 + \Delta t) n_{ij}(t_0) \tag{S11}$$

where N_M is the number of monomers inside the heterochromatin sections and $n_{ij}(t)$ is an elements of the neighbourhood matrix at time t:

$$\mathbb{N} = \begin{pmatrix} 0 & n_{12} & n_{13} & \dots & n_{1N_M} \\ n_{21} & 0 & n_{23} & \dots & n_{2N_M} \\ \vdots & \vdots & \vdots & \ddots & \vdots \\ n_{N_M1} & n_{N_M2} & n_{N_M3} & \dots & 0 \end{pmatrix}. \tag{S12}$$

The value of $n_{ij}(t)$ tracks which monomers are in contact with a particular monomer as follows:

$$n_{ij}(t) = \begin{cases} 0 & \text{for} \quad i = j, \\ 1 & \text{if} \quad d_{ij}(t) < R_D, \\ -1 & \text{if} \quad d_{ij}(t) \geq R_D. \end{cases} \tag{S13}$$

Here $d_{ij}(t)$ is the distance between ith and jth monomer; R_D is the radius of the droplet. This construction is shown schematically in Fig. S10(A) (Supporting Information). Note that $\mathbb{N}$ is invariant under $\mathbf{E}(n)$ group operations on the polymer as a whole. Therefore, any global rotation or translation of the polymer chain will not affect the correlation; only local rearrangements contribute to loss in correlation.

We have simulated two polymer chains with half and fully methylated heterochromatin blocks. The decay in C_{NN} in Fig. S10(B) (Supporting Information) indicates significant rearrangements of the polymer with time. The local rearrangements are slower for the fully methylated case but in both cases the dramatic decay in C_{NN} indicates that there is a very dynamic rearrangement of the polymer inside the droplet.

S4 Kinetic Monte Carlo scheme for methylation reactions

After the half-methylated co-polymer induces a droplet, we start with the chemical reactions. We perform methylation attempts on a randomly picked subset of the monomers through a Kinetic Monte Carlo (KMC) scheme, followed by a short MD run for re-equilibration. We call this entire step a Monte Carlo sweep. A single cell generation consists of a certain number of MC sweeps. Here, we formulate the kinetics of the reaction scheme using an extended version of the Gillespie algorithm [8], a method for simulating chemical reactions.

Let s_i be the state of a monomer that consists of its type $\mathcal{T}$, methylated (M) or unmethylated (U), and any other associated variables that are "instantaneous" during an MC sweep (e.g. the HP1 particle counts $n(s_i)$, computed right after an MD run). From state s_i, the enzymatic reaction $\mathcal{E}(s_i)$ can take the ith monomer, if in state U, to a methylated state (U $\longrightarrow$ M). Since, for simplicity, it is assumed here that methylation is irreversible, there is no backflow to s_i. The propensity (rate) or catalytic rate for monomer i in state s_i to get methylated is assumed to depend exponentially on the number $n(s_i)$ of HP1s in contact with:

$$a(s_i) = k_0 \, e^{-\, n(s_i)\, \epsilon_m} \tag{S14}$$

with $\epsilon_m < 0$. There is a delta function implied in the propensity as $\delta(\mathcal{T} - U)$, which excludes a methylation reaction of an already methylated monomer (for simplicity, we do not consider di- and

trimethylations here).

The stochastic master equation for the probability $p(s_i;t)$ to still be in state s_i at time t is given by:

$$\frac{d}{dt}p(s_i;t) \;=\; -a(s_i)\,p(s_i;t). \tag{S15}$$

For the initial condition $p(s_i;0) = 1$ this is solved by

$$p(s_i;t) \;=\; \exp\big[-a_i(s)\,t\big] \;=\; S(s_i;t) \tag{S16}$$

i.e., $S(s_i;t)$ is the survival probability for the nucleosome in state s_i. The associated escape or methylation probability follows as

$$p_m(t) \;= 1 - \; S(s_i;t) = \; 1 - \exp\big[-a_i(s)\,t\big]. \tag{S17}$$

Discrete-time "sweep" formulation with multiple independent methylations

Each MC sweep with duration Δt evaluates a randomly selected batch B of k monomers (a subset of all monomers) in parallel. Within a sweep, rates are frozen (computed at sweep start), and each monomer $i \in B$ is methylated independently with per-sweep methylation probability

$$p_m \;=\; 1 - e^{-a(s_i)\,\Delta t}. \tag{S18}$$

The probability that no methylation occurs in the sweep is the product of survivals:

$$\text{Pr(no event in sweep)} \;=\; \prod_{i\in k}(1-p_i) \;=\; \exp\Big(-\sum_{i\in k} a(s_i)\,\Delta t\Big). \tag{S19}$$

The batch escape rate λ_k is given by $\lambda_k = \sum_{i\in k} a(s_i)$. From this follows the per-sweep escape probability or methylation probability:

$$\alpha_k \;=\; 1 - \exp\big(-\lambda_k\,\Delta t\big). \tag{S20}$$

Gillespie's algorithm advances one reaction at a time, which makes it very slow when propensities are high or when dealing with large systems. Therefore, a faster, approximate method is desirable. Next, we will discuss an algorithm that processes multiple reactions simultaneously under certain approximations within a fixed time interval, thereby speeding up the computation.

τ-leap KMC scheme

We consider N different reactant species $\{s_1, s_2, ..., s_N\}$ that can undergo reactions in Y different reaction channels $\{R_1, R_2, ..., R_Y\}$. The τ-leaping idea accelerates the simulation by "freezing" propensities over a short interval $[t, t+\tau]$ and leaping the state forward by sampling how many times each channel fires in that interval. Following the original Poisson τ-leap algorithm [9, 10], assuming $a(s_i; R_j)$ (the propensity of reaction R_j on reactant s_i) is approximately constant between the time interval $[t, t+\tau]$, we draw the number of possible reactions that can happen from a Poisson distribution:

$$K_{ij} \sim \text{Poisson}\big(a(s_i; R_j), \tau\big). \tag{S21}$$

The probability that the channel fires at least once in the leap is $1 - \exp\big[-a(s_i; R_j)\,\tau\big]$ and the probability that nothing fires at all is $\exp\big[-a(s_i; R_j)\,\tau\big]$. Accuracy requires that propensities do not change appreciably during the leap; the time interval should be small enough to ensure this.

A practical issue with Poisson τ-leap is the possibility of negative populations if a Poisson draw consumes more molecules than are available. To remedy this situation, a modified version of this framework, the so-called called Bernoulli τ-leap [11], can be used. Here, one sets a limit on the maximum number of reaction channels that can fire during the time interval, thus avoiding the 'negative population issue'.

In our methylation scheme, this cap on maximum firing of a reaction channel comes naturally. We have only one type of methylation reaction, and a single monomer can methylate at most one time. To map the τ-leap scheme on our enzymatic reaction framework, we have, during an MC sweep, k reactant species (or monomer states), $\{s_1, s_2, ..., s_k\}$, and only R_1-type reactions that can occur only once. Therefore, the probability of a reaction is similar to the escape probability given in Eq. (S17):

$$p_m(s_i) = 1 - \exp\{-a(s_i)\tau\} = 1 - \exp\{-k_0\, e^{-n(s_i)\,\epsilon_m}\tau\} = 1 - \exp\{-p_m^0\, e^{-n(s_i)\,\epsilon_m}\} \tag{S22}$$

where $p_m^0 = k_0\tau$ is the intrinsic catalytic probability of the enzyme. Therefore, the definition of k_0 is the methylation probability per unit time in the absence of HP1s, $k_0 = -\frac{1}{\tau}\ln(1 - p_m(0))$.

In practice, we implement the following steps in our simulation:

1. Select a subset of monomers randomly with uniform distribution.

2. Count the HP1 particles in contact with a monomer from this subset.

3. Generate a random number α from a uniform distribution between $[0, 1]$.

4. Change the particle type to M if $\alpha < \mathbf{min}\Big(1, [1 - \exp\{-p_m^0\, e^{-n(s_i)\,\epsilon_m}\}]\delta(\mathcal{T} - U)\Big)$; else reject it.

5. Repeat step 2 to 4 for all k monomers.

When the methylation probability is very small, it can be approximated as

$$p_i \;= 1 - \exp\{-k_0\, e^{-n(s_i)\,\epsilon_m}\tau\} \;\approx\; k_0\, e^{-n(s_i)\,\epsilon_m}\tau = p_m^0\, e^{-n(s_i)\,\epsilon_m}. \tag{S23}$$

S5 Quantification of degree of re-establishment of epigenetic marks

Here we describe the definitions of mismatches between the current epigenetic sequence and a target sequence that the system is attempting to reach through methylation reactions. These mismatches are used in Figs. 5(B) and 8. We first define the target sequence. At the beginning of the first cell cycle we start from the 50 monomers long blocks of heterochromatin. After removing half of the epigenetic marks, blocks might have shrunk, as some of the nucleosomes at the boundaries have lost their marks. Our re-establishment scenario has no way to determine the exact locations of the original hetero-/euchromatin boundaries. We thus define the slightly smaller domains, including the outermost still methylated nucleosomes, as new heterochromatin domains. This sequence with all its defects inside the new heterochromatin domains methylated and all nucleosomes in the euchromatin domains unmethylated is our target sequence. Also in each following cell cycles we use this rule to set a new target sequence. We define now the heterochromatin mismatch as

$$\xi_H(t_M) = \frac{n_T - n_H(t_M)}{n_T} = \frac{\Delta n_H(t_M)}{n_T} \tag{S24}$$

where n_T is the total number of marked nucleosomes in the target sequence of a given cell cycle. Moreover, $n_H(t_M)$ is defined as the number of methylated nuclesomes inside the heterochromatin

domains of the target sequence which is a function of the MC sweep t_M. This quantity tracks how the defects of missing epigenetic marks are closed over time.

In addition, we have two more mismatches. These keep track of the methylation of "wrong" nucleosomes (i.e. nucleosomes in the heterochromatin domains of the target sequence): the mismatch ξ_b due to the growth of heterochromatin into euchromatin (the boundary growth) and the mismatch ξ_e that results from the spontaneous formation of epigenetic marks on nucleosomes inside the euchromatin domains (but not in direct contact to the current boundaries between eu- and heterochromatin). Specifically

$$\xi_b\left(t_M\right)=\frac{\Delta n_b\left(t_M\right)}{n_T}, \quad \xi_e\left(t_M\right)=\frac{\Delta n_e\left(t_M\right)}{n_T}. \tag{S25}$$

Here $\Delta n_b\left(t_M\right)$, $\Delta n_e\left(t_M\right)$ again count errors by comparing with the target sequence.

S6 Heterochromatin mismatch decay curve fit

We fit the data for heterochromatin mismatch decay at different values of distribution bias. The equation we fit to reads

$$\xi_H = {\xi_H}^0 \exp\left(-(t_M/\tau)^\nu\right) \tag{S26}$$

(also given by Eq. 6). In order to get a consistent fit, we find the region where ν remains nearly constant by evaluating the slope of the plot $\log(t_M)$ vs $\log(-\log(\xi_H/\xi_H^0))$, assuming $\xi_H^0 = 50$. We find the mid-range is ideal for a stretched exponential fit. The fitted parameters are listed below.

Distribution bias (p_b)	ξ_H^0	τ	ν
0.50	48.94	69.08	1.144
0.40	50.86	83.08	0.949
0.20	52.01	140.66	0.719
0.0	47.58	375.34	0.722

S7 Average restoration score

In order to quantify the efficacy of the restoration mechanism over many cell generations (MCG), we introduce a metric called the average restoration score $\langle \rho_R \rangle$. In our system, each monomer has two possible states, $\mathcal{T}$, methylated (**M**) or unmethylated (**U**). The sequence of monomers consisting of three fully methylated blocks of length 50, alternating with four euchromatin blocks of the same length, is our target sequence, which we denote by S_I. If G consecutive cell generations are simulated, then the final product sequence from the Gth generation is referred to as S_F. The average restoration score of the ith nucleosome is then defined as

$$\langle \rho_R \rangle_i = \left\langle \delta(\mathcal{T}^i_{S_F} - \mathcal{T}^i_{S_I}) - \delta(\mathcal{T}^i_{S_I} - \mathbf{U}) \right\rangle_E \tag{S27}$$

where $\delta(0) = 1$ and E stands for the total number of ensembles, a set of independent MCG simulations. The metric, inside the $\langle \ldots \rangle$, gives a positive score (+1) when a methylated nucleosome is restored as methylated ($\mathbf{M} \rightarrow \mathbf{M}$), a negative score ($-1$) when an unmethylated nucleosome is transformed to the methylated state ($\mathbf{U} \rightarrow \mathbf{M}$) and a neutral score (0) when an unmethylated nucleosome remains unmethylated ($\mathbf{U} \rightarrow \mathbf{U}$). Average restoration scores for various parameters are provided in Fig. S7.

In addition, we use the average restoration score, Eq. S27, to quantify the heterochromatin domain restoration efficiency based on the cumulative distribution function

$$\Phi_R(x) = \frac{1}{N_H} \sum_{i \in D} \Theta(\langle \rho_R \rangle_i - x), \qquad 0 \le x \le 1, \tag{S28}$$

where N_H is the number of heterochromatin monomers, and D is the set of monomers that belong to the heterochromatin domains. $\Theta(z)$ denotes the Heaviside step function with $\Theta(z) = 1$ for $z \ge 0$ and 0 otherwise. $\Phi_R(x)$ thus provides the cumulative fraction of heterochromatin monomers that remain methylated after G cell generations. This is shown for $G = 50$ and different cell cycle cutoff times in Fig. S8.

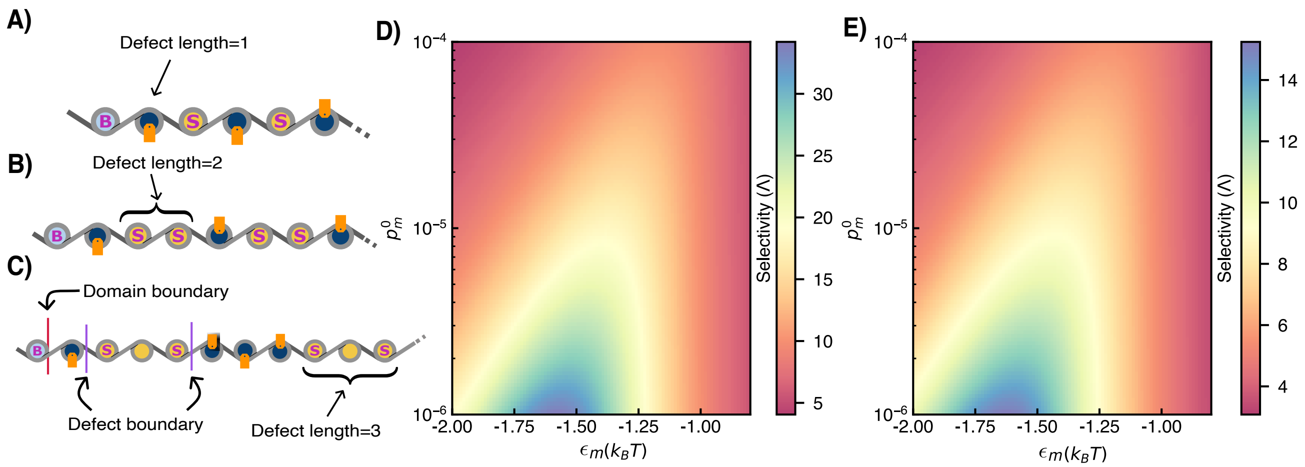

Figure S1: **Selectivity variation with defect length:** Definition of monomer sets **S** and **B** in Eq. 5 for different defect lengths: (A) one, (B) two and (C) three nucleosomes. (D) Selectivity map for alternating sequence of blocks of two marked and two unmarked nucleosomes. (E) Same as (D) but for block size three. Selectivity values are strongly reduced compared to the case of block size one, Fig. 4(B).

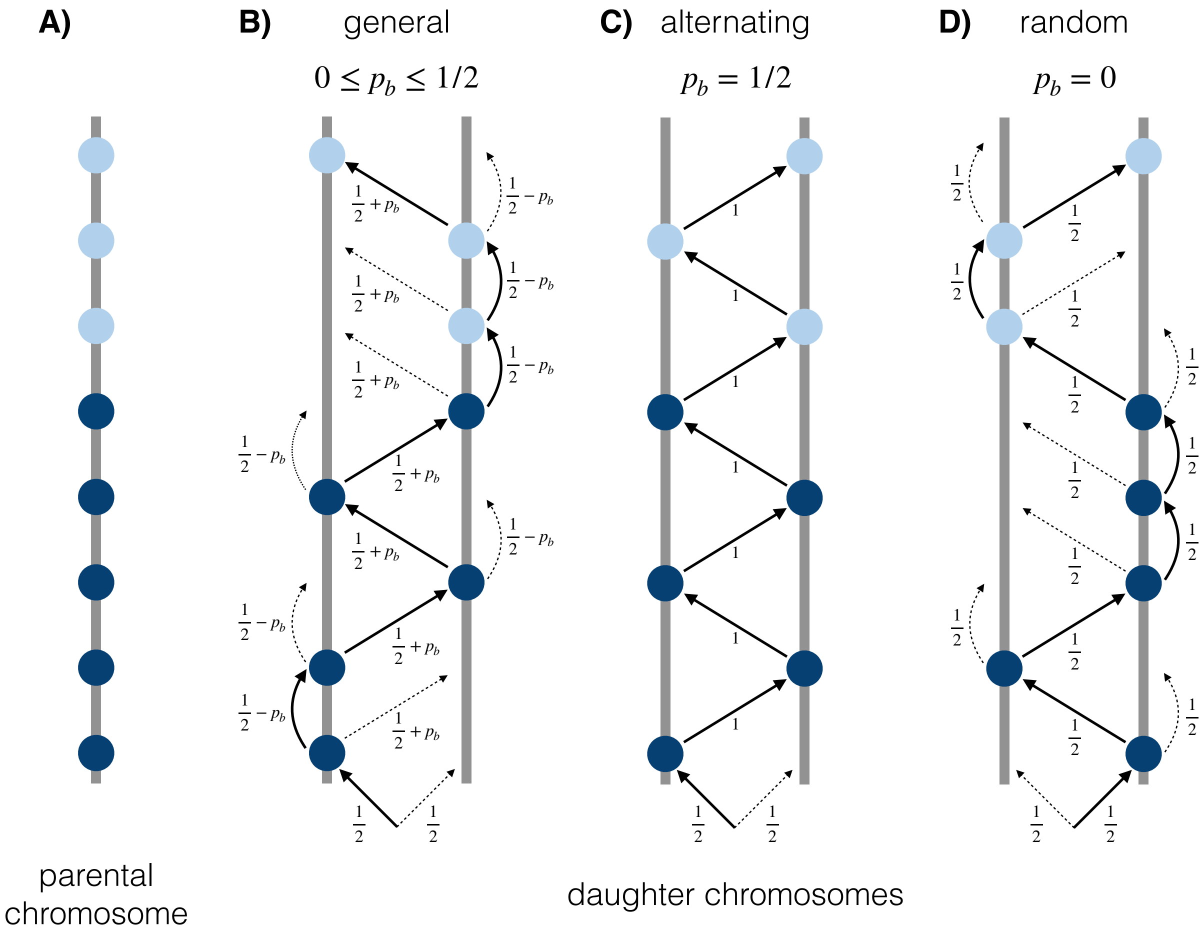

Figure S2: **Epigenetic dilution scheme:** (a) Original sequence of epigenetically marked nucleosomes. (b) Scheme for distributing the nucleosomes between the two daughter cells for an arbitrary value of the distribution bias p_b, $0 \le p_b \le 1$. Starting from the bottom, we choose the first nucleosome to go to either DNA copy with equal probability $1/2$. From then on, the next nucleosome is chosen to stay on the same DNA molecules with probability $1/2 - p_b$ or to be distributed to the other molecule with probability $1/2 + p_b$.(c) Alternating case $p_b = 1/2$. (d) Completely random case $p_b = 0$.

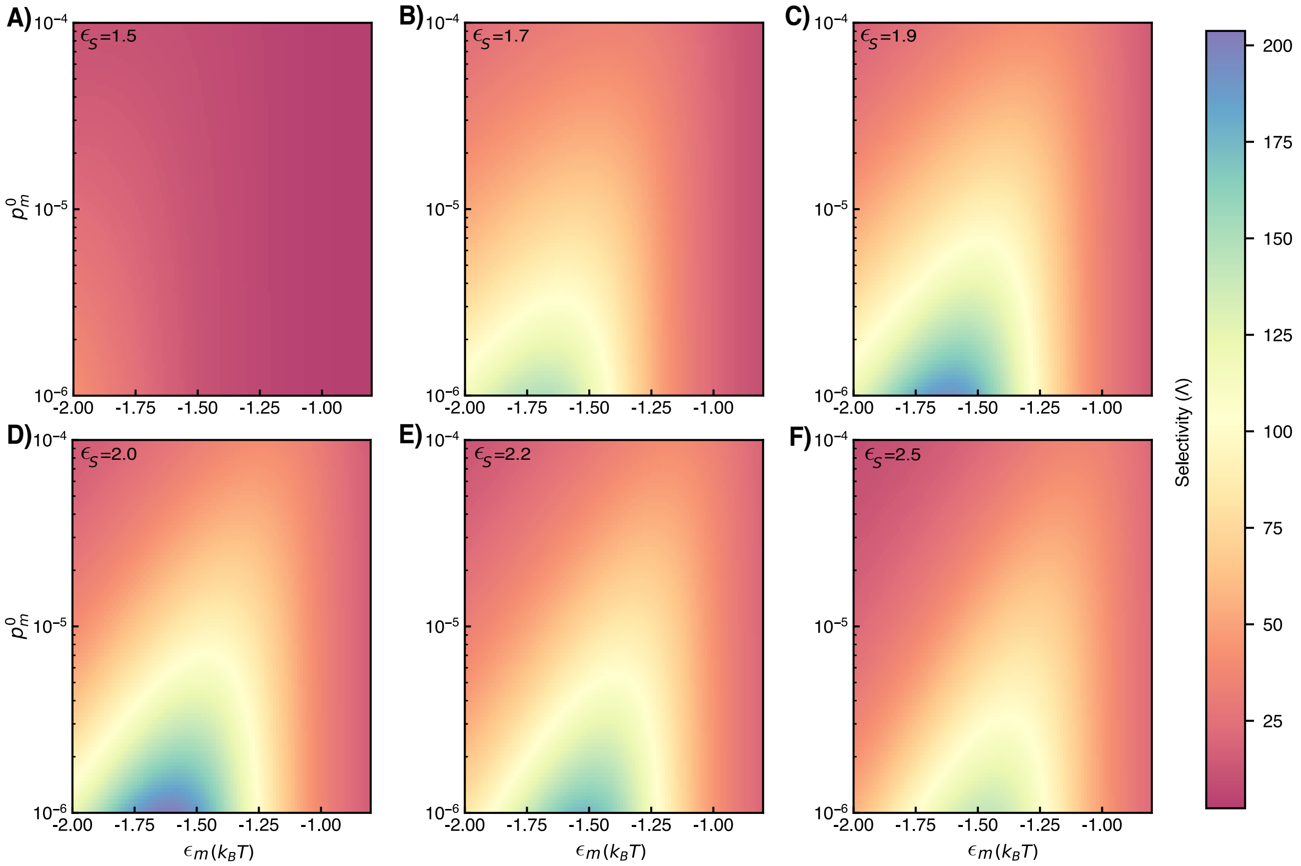

Figure S3: **Selectivity variation with ϵ_S:** This panel shows maps of selectivity Λ in the p_m^0–ϵ_m parameter space for alternating heterochromatin sequences, as in Fig. 4(B), but for different values of attraction strength between labeled nucleosomes and HP1. Specifically: (A) $\epsilon_S = 1.5$, (B) $\epsilon_S = 1.7$, (C) $\epsilon_S = 1.9$, (D) $\epsilon_S = 2.0$ (identical to Fig. 4(B), (E) $\epsilon_S = 2.2$ and (F) $\epsilon_S = 2.5$. Each case leads to high selectivity values, except for $\epsilon_S = 1.5$. In this case, $\epsilon_S/2$ is close to the mixed state, see Fig. 3(E).

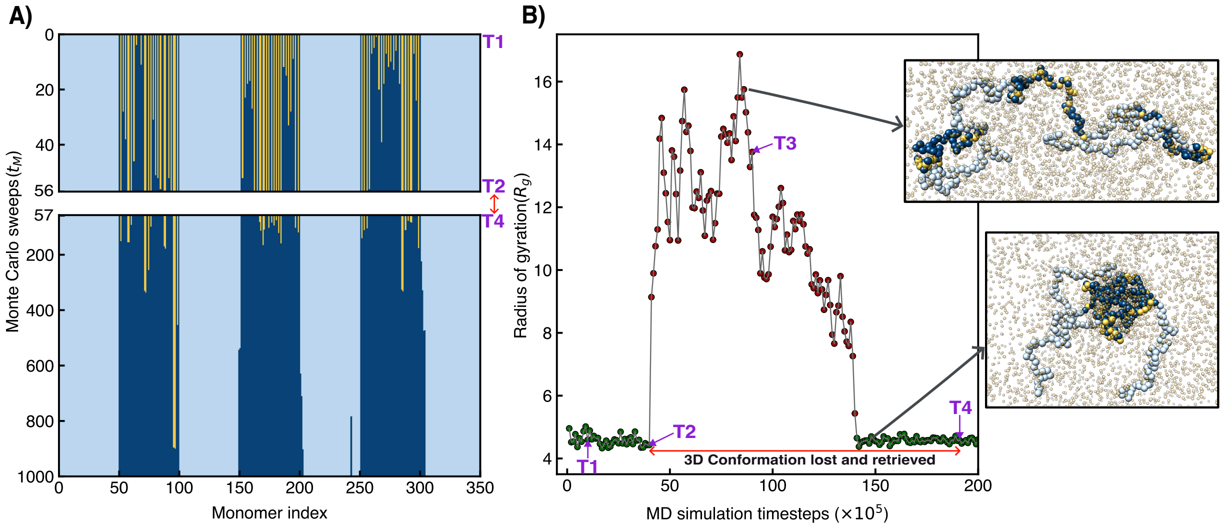

Figure S4: **Temporary disruption of condensate does not affect re-establishment of epigenetic marks:** (A) Development of epigenetic sequence from MC sweep 0, time $T1$, to MC sweep 56, time $T2$, and again, after disrupting the process, from MC sweep 57, time $T4$, onwards. The time $T2$ has been chosen when 75% of the heterochromatic monomers were methylated. (B) Radius of gyration of heterochromatin sections. At time $T2$, the attraction between the marked monomers and HP1 is switched off, and the structure expands, see snapshot on top. After switching on the attraction again at time $T3$, the polymer collapses again into a micelle, see bottom snapshot. The enzymatic reactions are switched off between times $T2$ and $T4$. We use here the same set of parameters ($\epsilon_m = -1.4$, $p_m^0 = 10^{-5}$) as in Fig. 5. Since these values give low methylation probabilities, we have used here the approximated expression given by Eq. S23.

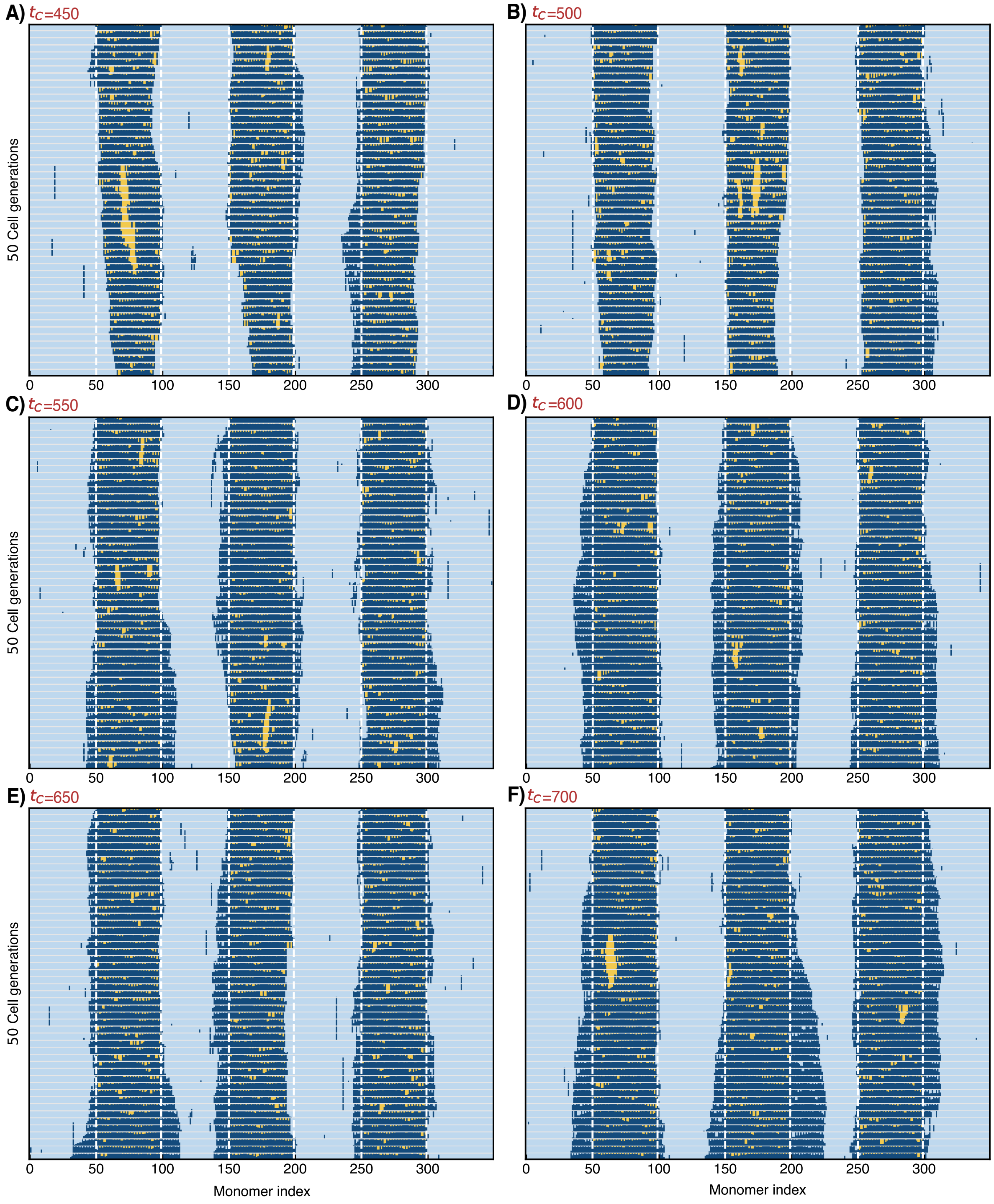

Figure S5: **Multiple cell-generation profiles for various cell cycle times:** Development of epigenetic sequences for 50 cell generations for different values of t_C, ranging from $t_C = 450$ up to 700.

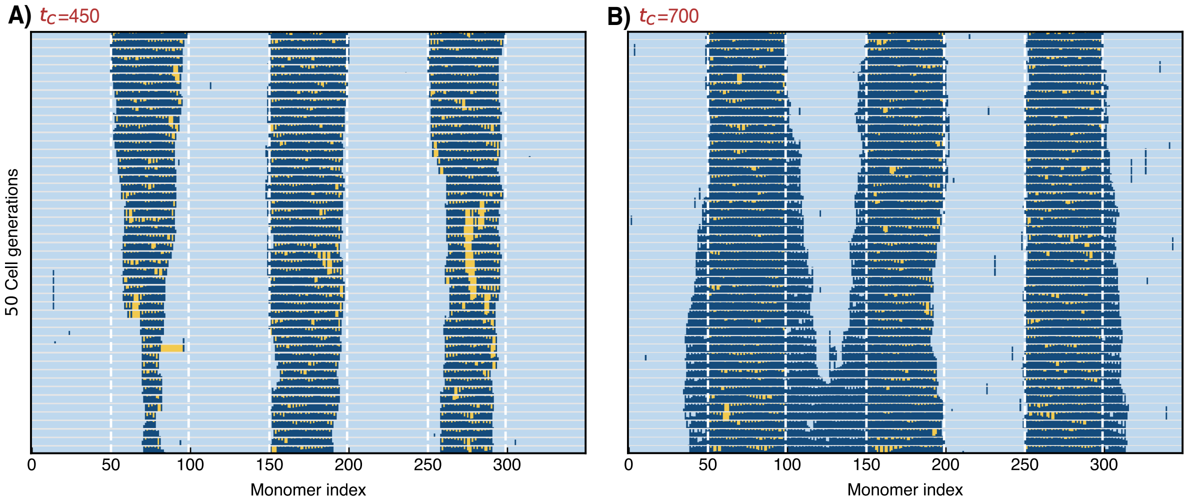

Figure S6: **Examples of 50 cell-generation profiles with substantial changes in epigenetic sequence:** (A) A simulation run with a cell cycle time of 450 where the leftmost domain almost disappeared. (B) An example with a cell cycle time of 700, where a new domain formed within euchromatin that eventually glued the neighboring domains together.

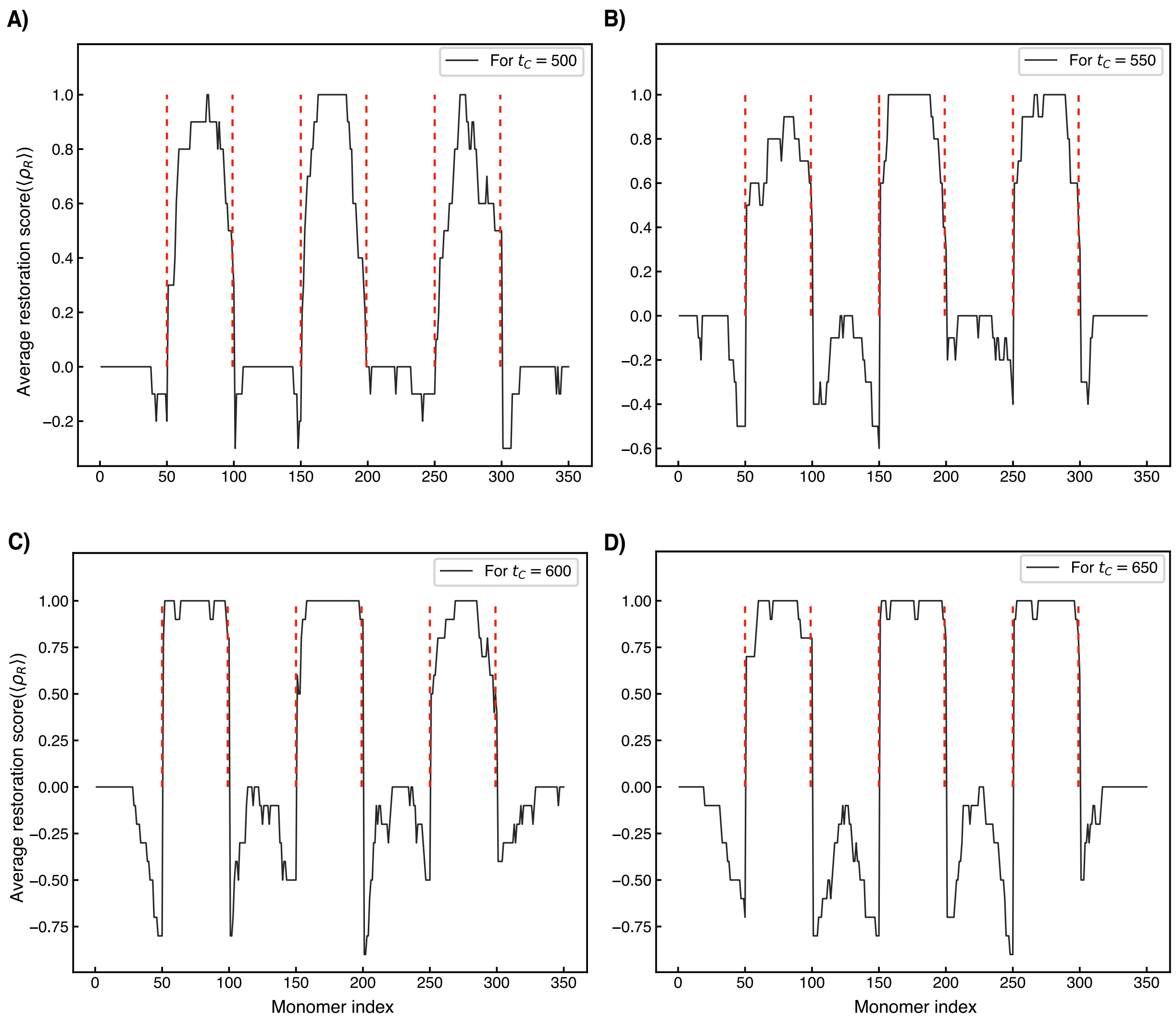

Figure S7: **Average restoration score of MCG sequences for different cell cycle cut-off times:** Restoration scores for each monomer, Eq. S27, after completion of 50 cell generations for different t_C-values: (A) 500, (B) 550, (C) 600 and (D) 650. Each plot is averaged over 10 independent MCG simulations with a fixed cell generation cut-off time. Perfect restoration leads to $\langle\rho_R\rangle = 1$ for heterochromatin monomers and to $\langle\rho_R\rangle = 0$ for euchromatin monomers. The original domain boundary positions are marked with red dashed lines.

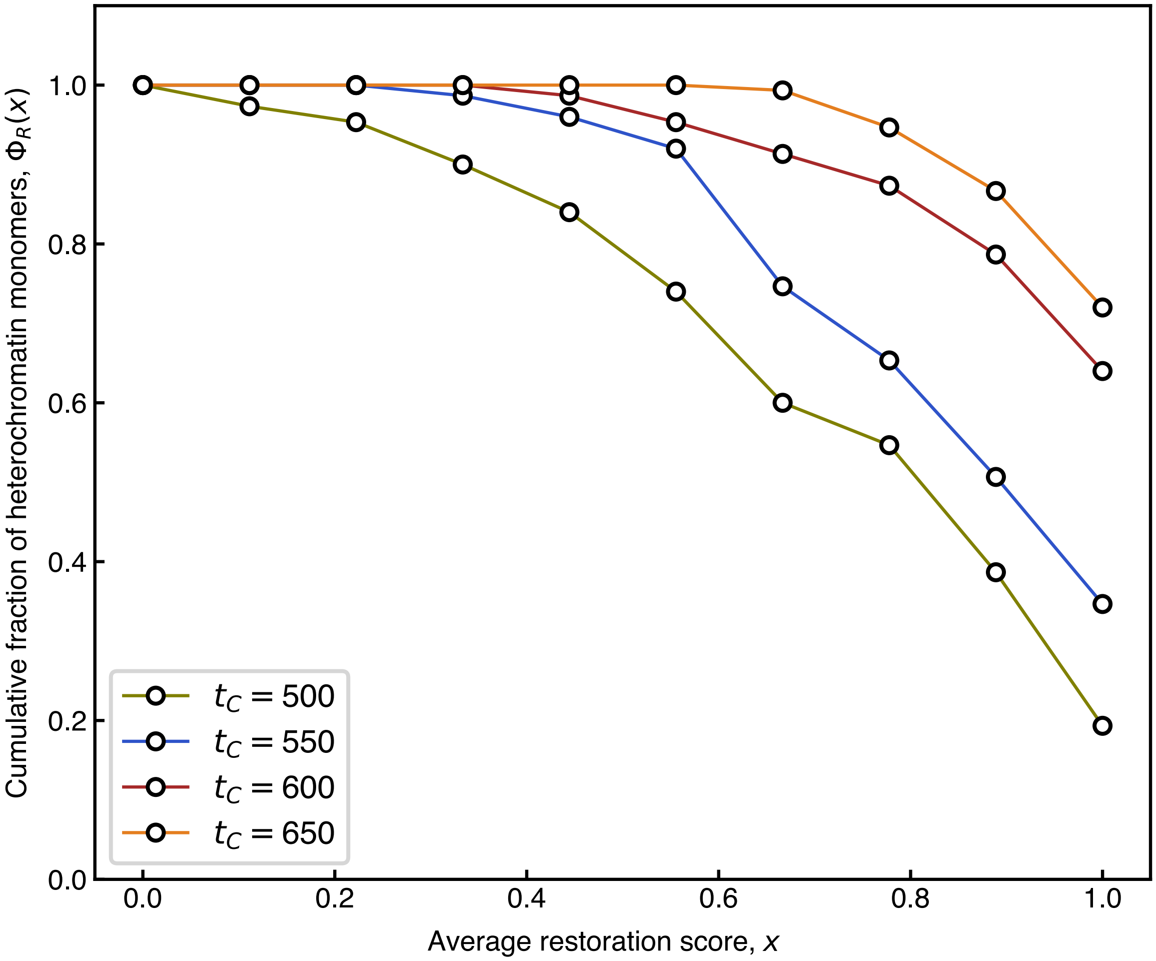

Figure S8: **Cumulative restoration score for heterochromatin domains:** Cumulative fraction of heterochromatin monomers (evaluated using Eq. S28) as a function of x. Inspecting e.g. the data point at $x = 0.8$ for the red curve ($t_C = 600$), one learns that at least 80% of the heterochromatic monomers got restored in in almost 90% of the simulation runs.

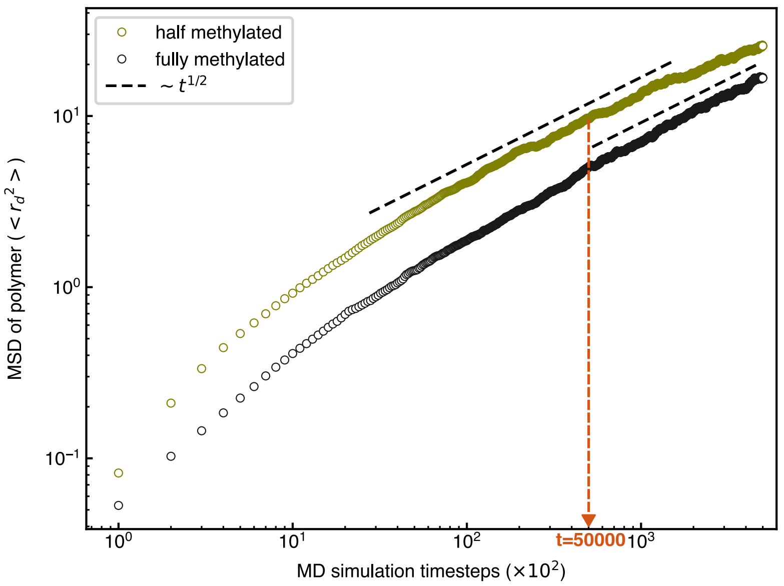

Figure S9: **Diffusion of heterochromatin inside droplet:** MSD of the monomers contained in the heterochromatin section as a function of the MD simulation time steps. Shown are curves for fully and half methylated heterochromatin. The lines indicate subdiffusive behaviour with exponent 1/2.

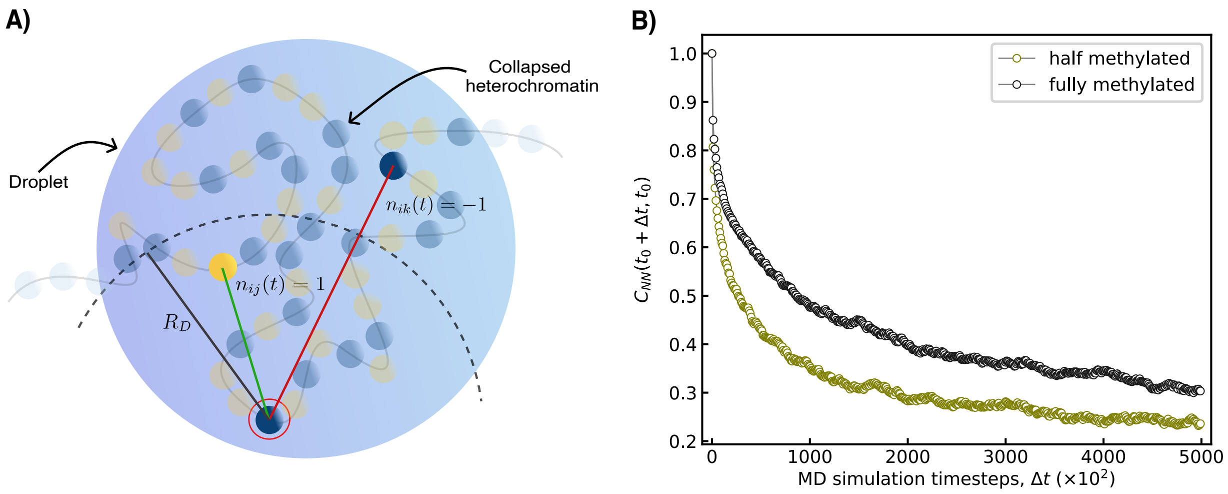

Figure S10: **Tracking the dynamic conformational rearrangement of heterochomatin:** (A) Schematics showing the mapping of the neighbours of a given monomer to a binary state. The neighbourhood radius is taken to be the radius of the droplet R_D. (B) Decay of neighbourhood-neighbourhood correlation with time for half and fully methylated chromosomes.

Supplementary References

[1] Kurt Kremer and Gary S. Grest. "Dynamics of entangled linear polymer melts: A molecular-dynamics simulation". In: *J. Chem. Phys.* 92.8 (1990), pp. 5057–5086.

[2] A. Galuschko and J.-U Sommer. "Co-nonsolvency response of a polymer brush: a molecular dynamics study". In: *Macromolecules* 52 (2019), pp. 4120–4130.

[3] H. Watanabe, N. Ito, and C.-K. Hu. "Phase diagram and universality of the Lennard-Jones gas-liquid system". In: *J. Chem. Phys.* 136 (2012), p. 204102.

[4] S. Plimpton. "Fast parallel algorithms for short-range molecular dynamics". In: *J. Comput. Phys.* 117 (1995), pp. 1–19.

[5] Sarah H. Sandholtz, Quinn MacPherson, and Andrew J. Spakowitz. "Physical modeling of the heritability and maintenance of epigenetic modifications". In: *Proc. Natl. Acad. Sci. USA* 117.34 (2020), pp. 20423–20429.

[6] J. A. Owen, D. Osmanović, and L. Mirny. "Design principles of 3D epigenetic memory systems". In: *Science* 382 (2023), eadg3053.

[7] H. Schiessel. *Biophysics for Beginners: A Journey Through the Cell Nucleus*. Singapore: Jenny Stanford Publ., 2022.

[8] Daniel T. Gillespie. "A general method for numerically simulating the stochastic time evolution of coupled chemical reactions". In: *J. Comput. Phys.* 22.4 (1976), pp. 403–434.

[9] Daniel T. Gillespie. "Stochastic simulation of chemical kinetics". In: *Annu. Rev. Phys. Chem.* 58 (2007), pp. 35–55.

[10] Daniel T. Gillespie. "Approximate accelerated stochastic simulation of chemically reacting systems". In: *J. Chem. Phys.* 115.4 (2001), pp. 1716–1733.

[11] Abhijit Chatterjee, Dionisios G. Vlachos, and Markos A. Katsoulakis. "Binomial distribution based τ-leap accelerated stochastic simulation". In: *J. Chem. Phys.* 122.2 (2005), p. 024112.
